# Supplementary material for: HiTE: a fast and accurate dynamic boundary adjustment approach for full-length transposable element detection and annotation
Source: Nat Commun. 2024 Jul 2;15:5573. doi: 10.1038/s41467-024-49912-8 (PMC11219922; doi:10.1038/s41467-024-49912-8)
Supplement: Supplementary file 1 — Supplementary Information [file 41467_2024_49912_MOESM1_ESM.pdf]

Supplementary Information for

HiTE: A fast and accurate dynamic boundary adjustment approach for full-length Transposable Elements detection and annotation

Hu *et al.*

Supplementary Figures

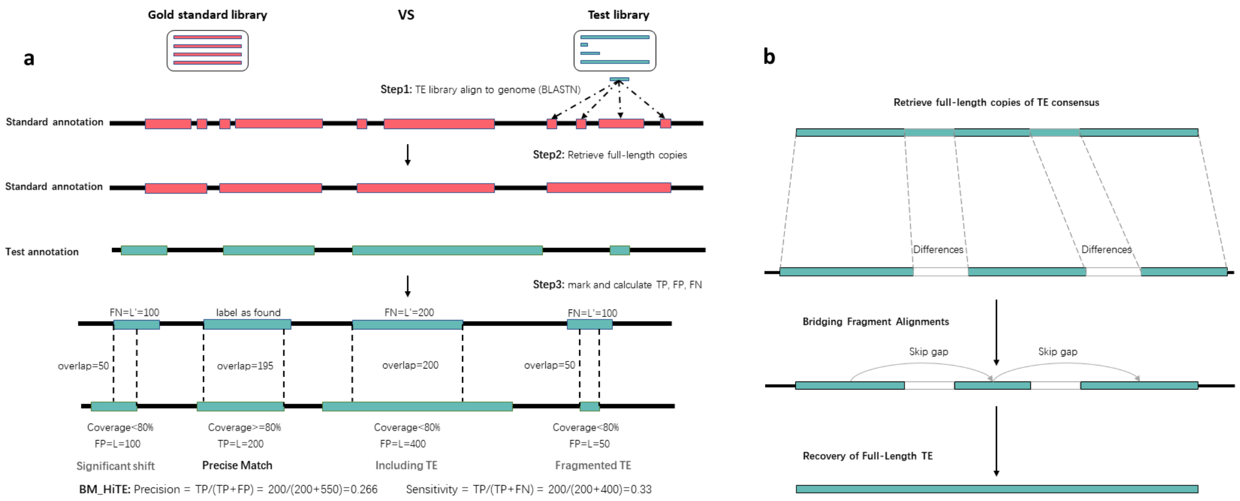

Supplementary Fig. 1 Schematic representation of the benchmarking method for HiTE (BM_HiTE). **a** The black line represents the genome, the pink bars indicate Repbase library annotations, and the blue bars represent test library annotations. Only when the length of the overlap between the test and Repbase TE sequence exceeds a threshold, such as 95% of their lengths respectively, it is considered a true positive. All others are considered as false positives, including alignments with significant shifts, longer sequences containing the true TE, and fragmented TEs. **b** Retrieving full-length copies from genome. Different copies of the same TE family often exhibit fragmented alignments. We employ our FMEA algorithm to connect fragmented alignments and recover full-length copies.

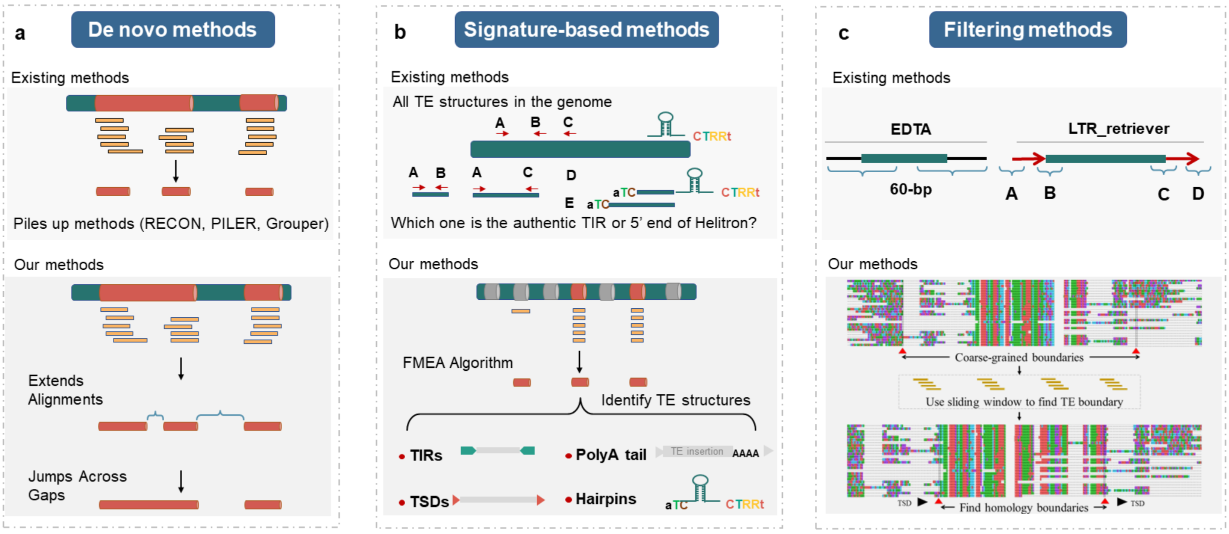

Supplementary Fig. 2 Contrast between HiTE and existing methods. **a** Existing methods cluster alignments, potentially leading to fragmented TE families due to the excessive fragmentation in alignments. Our FMEA algorithm is designed to connect and span insertions and gaps between alignment segments, aiming to preserve the integrity of TE instances. **b** The existing signature-based methods using the whole genome as input often lead to false positives due to the misleading nature of TE structural signals. HiTE performs signature-based identification on all potential TE candidate fragments generated by our FMEA algorithm. **c** Compared to the flanking sequence-based filtering rules for EDTA and LTR_retriever, we have developed a novel homology-based filtering method based on multiple sequence alignments, which can dynamically identify genuine TE boundaries. Green bars represent the genome, deep reddish-brown cylinders denote TEs, light yellow-orange bars indicate alignments, red arrows symbolize terminal inverted/direct repeats, and gray cylinders represent false-positive sequences with TE structures.

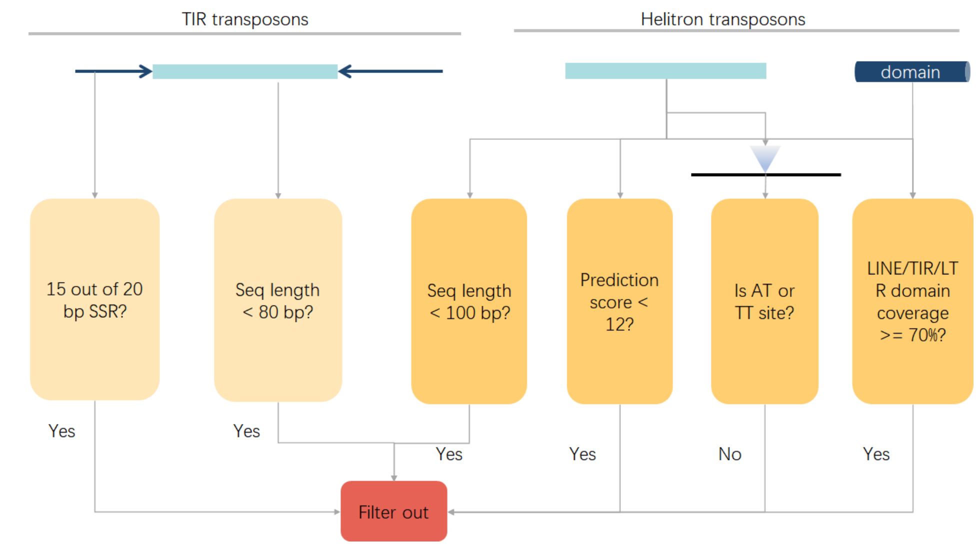

Supplementary Fig. 3 The illustration of EDTA filtering rule. EDTA filters out TIR elements carrying significant SSR sequences at either end. It also screens for short sequences, such as TIR transposons less than 80 bp and Helitron transposons less than 100 bp. Additionally, EDTA excludes Helitron elements with a HelitronScanner prediction score below 12 and those that do not insert into AT or TT sites. Helitron elements with over 70% length coverage with non-Helitron domains are also filtered out by EDTA.

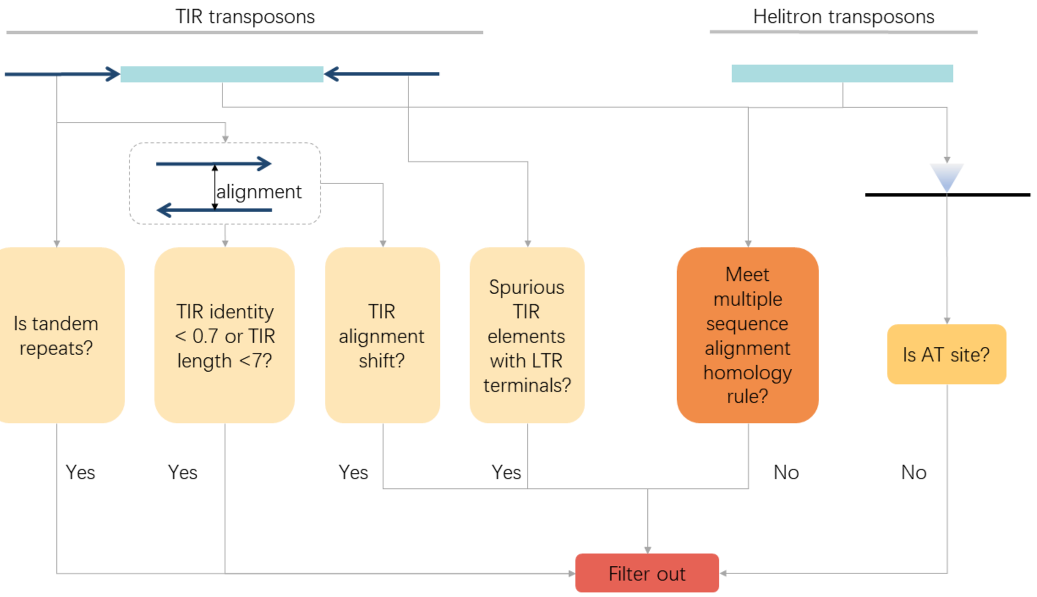

Supplementary Fig. 4 The illustration of HiTE filtering rule. HiTE applies various filtering criteria, including removal of tandem repeats, sequences with TIR identity <0.7 or TIR length <7, those with terminal inverted repeats exhibiting offset alignments, sequences with more than 95% alignment coverage with LTR or LTR internal regions due to occasional TIR-like structures, sequences that do not adhere to the homology rules of multiple sequence alignment, and Helitron candidates that do not insert into AT sites.

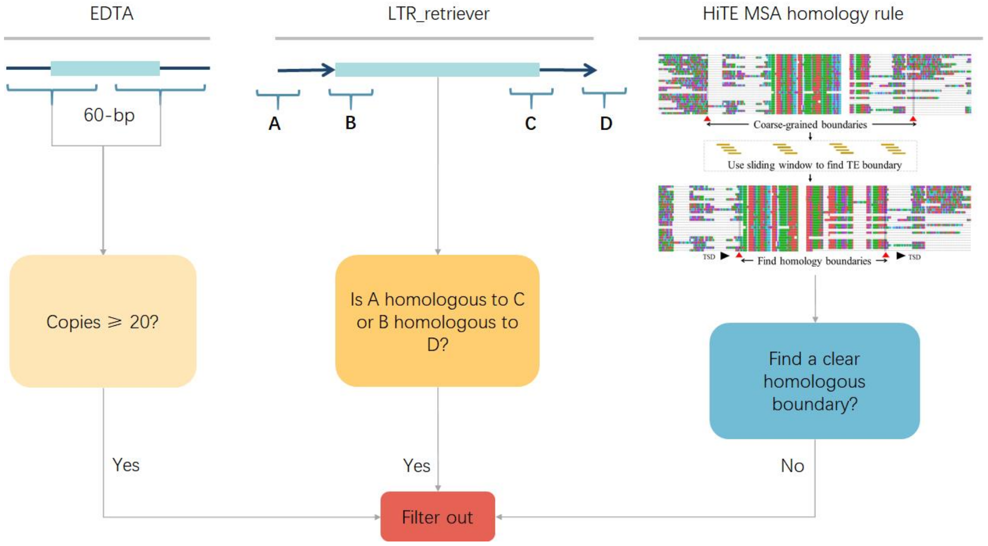

Supplementary Fig. 5 The illustration of filtering rule based on flanking sequences. EDTA considers a copy number of 20 or more in the 60-bp flanking region at either end as a false positive. LTR_retriever considers an LTR-RT candidate as a false positive if any flanking sequences of the direct repeat are aligned. HiTE considers a sequence as a false positive if clear homologous boundaries cannot be identified from the aligned multiple copies.

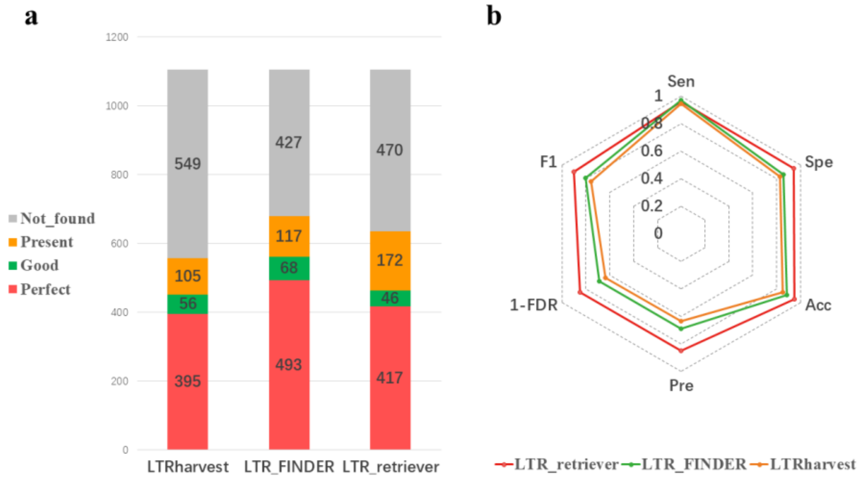

Supplementary Fig. 6 Performance comparison of LTR based on *O. sativa*. **a, b** Performance comparison of LTR annotators based on BM_RM2 and BM_EDTA, respectively. BM_RM2: Benchmarking method of RepeatModeler2; BM_EDTA: Benchmarking method of EDTA; Sen: Sensitivity; Spe: Specificity; Acc: Accuracy; Pre: Precision; FDR: False discovery rate.

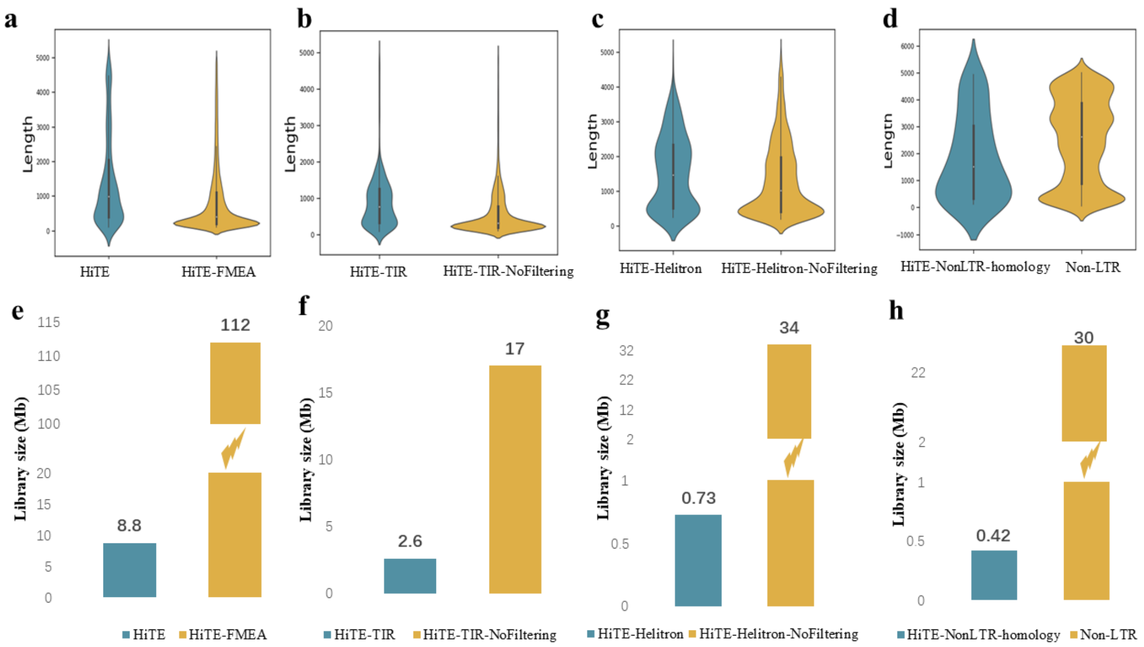

Supplementary Fig. 7 Length and size of candidate sequences before and after processing based on *O. sativa*. **a, e** Distribution of length and library size differences of candidates identified with or without fine-grained TE detection and false-positive filtration. The HiTE and HiTE-FMEA libraries contain 4,681 and 159,583 sequences, respectively. **b, f** Distribution of length and library size differences of TIR elements identified with or without false-positive filtration. The HiTE-TIR and HiTE-TIR-NoFiltering libraries contain 2,224 and 23,582 sequences, respectively. **c, g** Distribution of length and library size differences of TIR and Helitron elements identified with or without false-positive filtration. The HiTE-Helitron and HiTE-Helitron-NoFiltering libraries contain 371 and 18,861 sequences, respectively. **d, h** Distribution of length and library size differences between HiTE-NonLTR-homology and the known non-LTR library. The HiTE-NonLTR-homology and Non-LTR libraries contain 148 and 8,899 sequences, respectively. The boxes inside the violin plots indicate the 50th percentile (middle line), 25th, and 75th percentile (box).

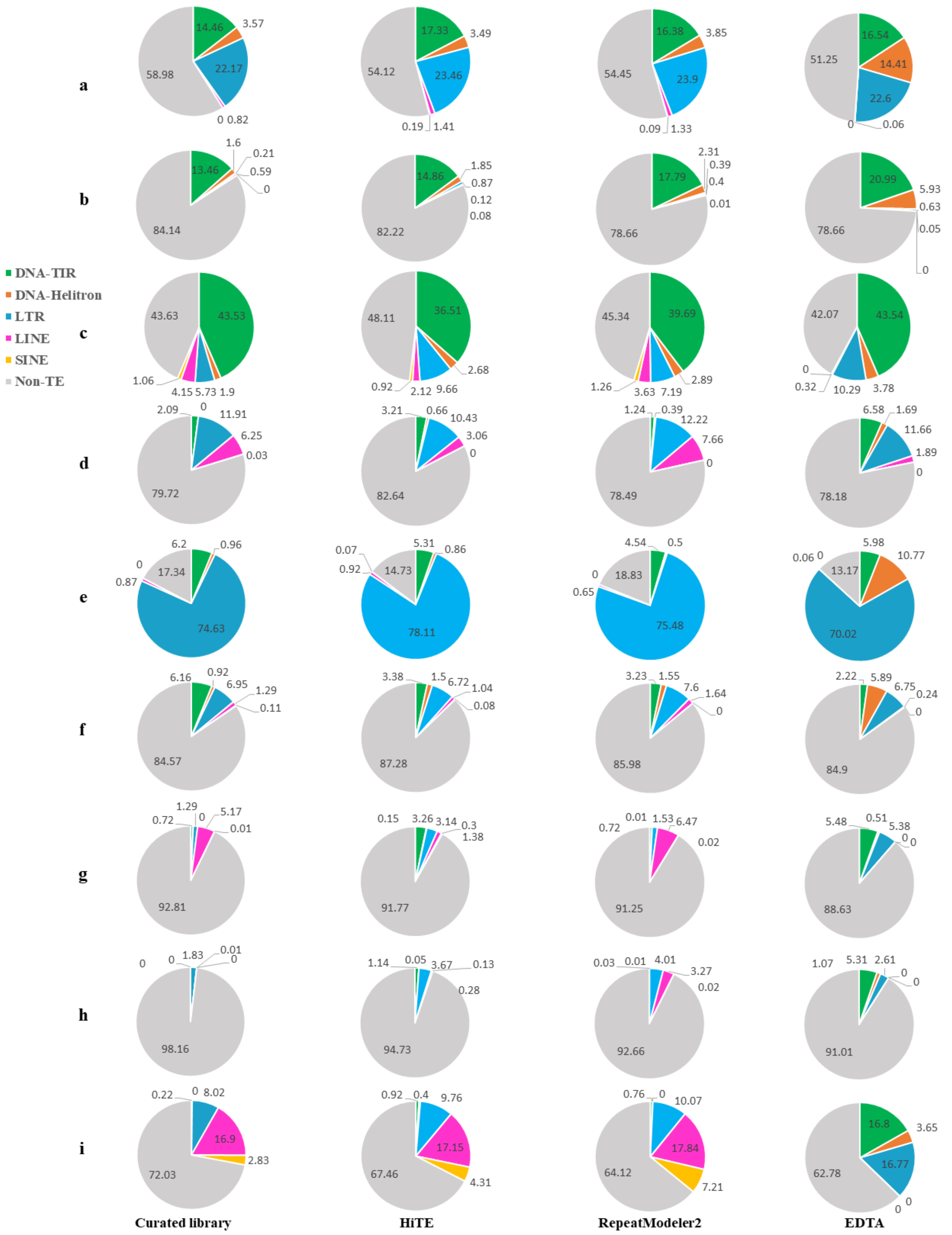

Supplementary Fig. 8 Percentage of the genome masked by each major subclass using five different types of TE libraries generated by manual curation, HiTE, RepeatModeler2, and EDTA, respectively. **a, b, c, d, e, f, g, h, and i** Percentage of the *O. sativa*, *C. briggsae*, *D. rerio*, *D. melanogaster*, *Z. mays*, *A. thaliana, G. gallus, T. guttata,* and *M. musculus* genome masked by these TE libraries, respectively. Note that the library generated by RepeatScout is not classified and therefore not presented.

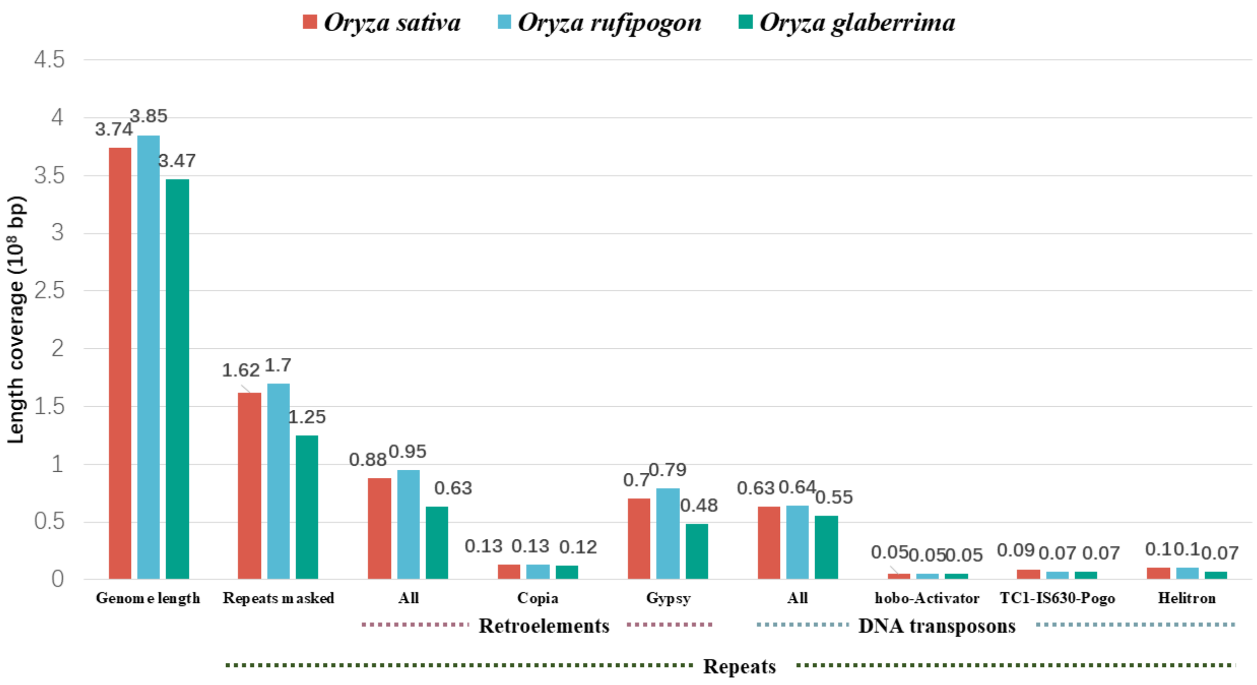

Supplementary Fig. 9 The length coverage distribution of different types of transposons based on the analysis of three types of rice species using HiTE.

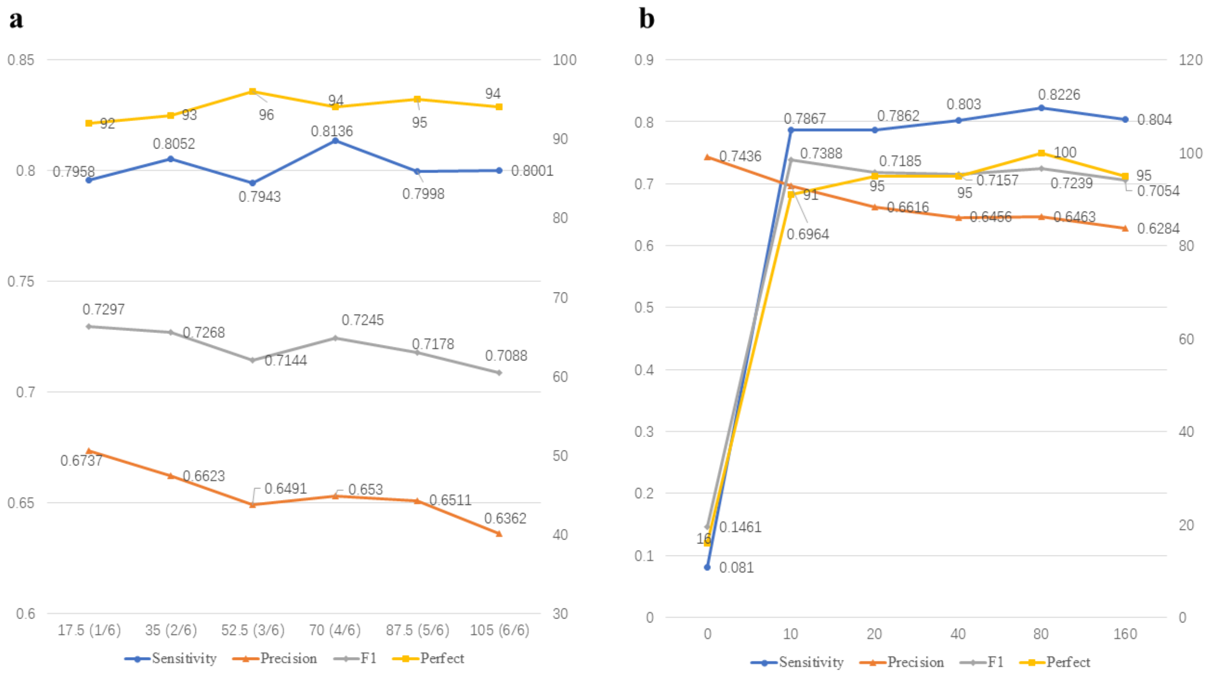

Supplementary Fig. 10 Evaluation of the impact of HiTE parameters on performance assessed by BM_RM2 and BM_HiTE (coverage>0.95) in *C. briggsae*. **a** Trend of performance change with the change of parameter *chunk_size*. The x-axis coordinates divide the genome into equal proportions from small to large. **b** Trend of performance change with the change of parameter *flanking_len*.

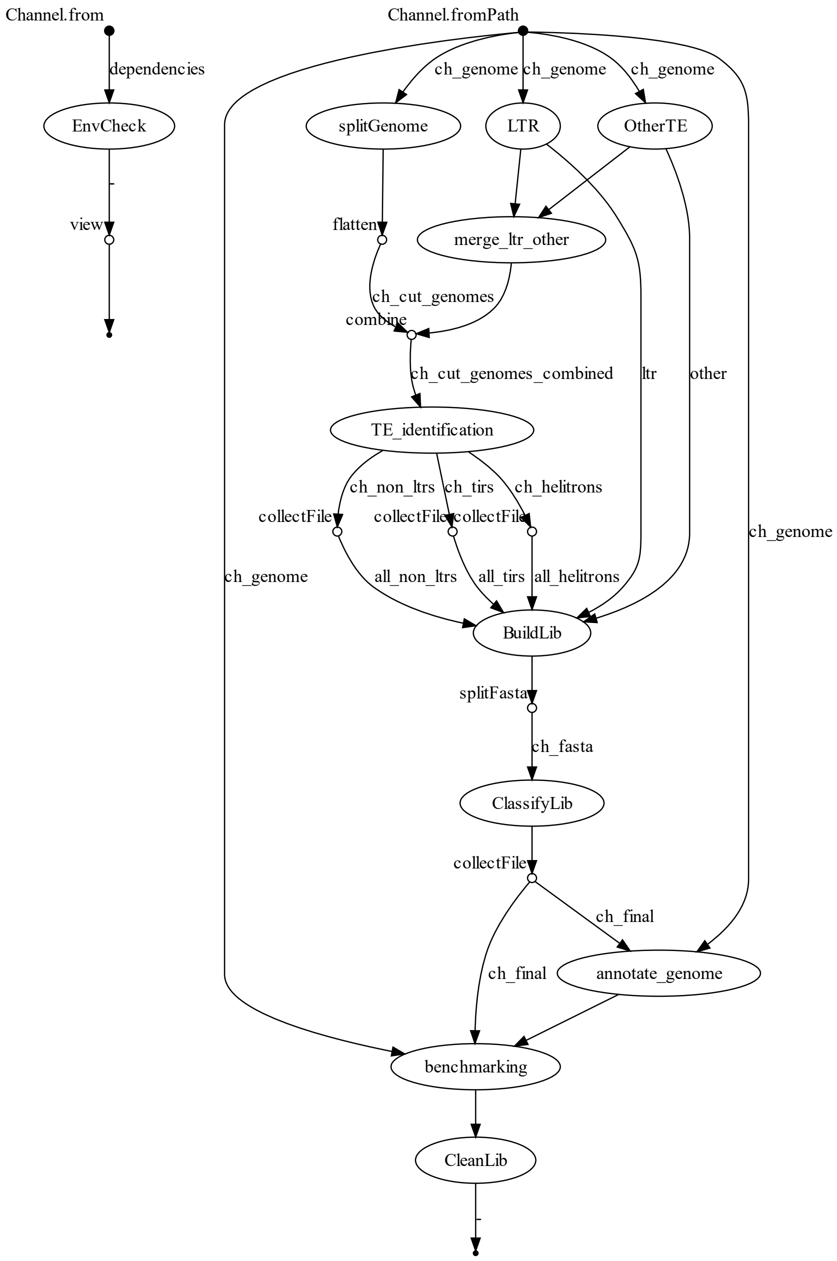

Supplementary Fig. 11 The NextFlow implementation flow chart of HiTE. EnvCheck: Checking the software dependencies required; splitGenome: Segmenting the genome into independent chunks suitable for parallel processing across multiple nodes; LTR: Detecting LTR transposons based on the input genome; OtherTE: Identifying non-LTR transposons using homology searches; merge_ltr_other: A preliminary step ensuring the execution of LTR and homology-based non-LTR transposons before the TE identification process; TE_identification: Identifying non-LTR, TIR, and Helitron elements. BuildLib: Unwrapping nested TEs and generating TE consensus sequences; ClassifyLib: Classifying TE consensus sequences using a parallel version of RepeatClassifier; annotate_genome: Annotating the genome using identified TE families; benchmarking: Performing benchmarking replication. CleanLib: Removing unnecessary temporary files.

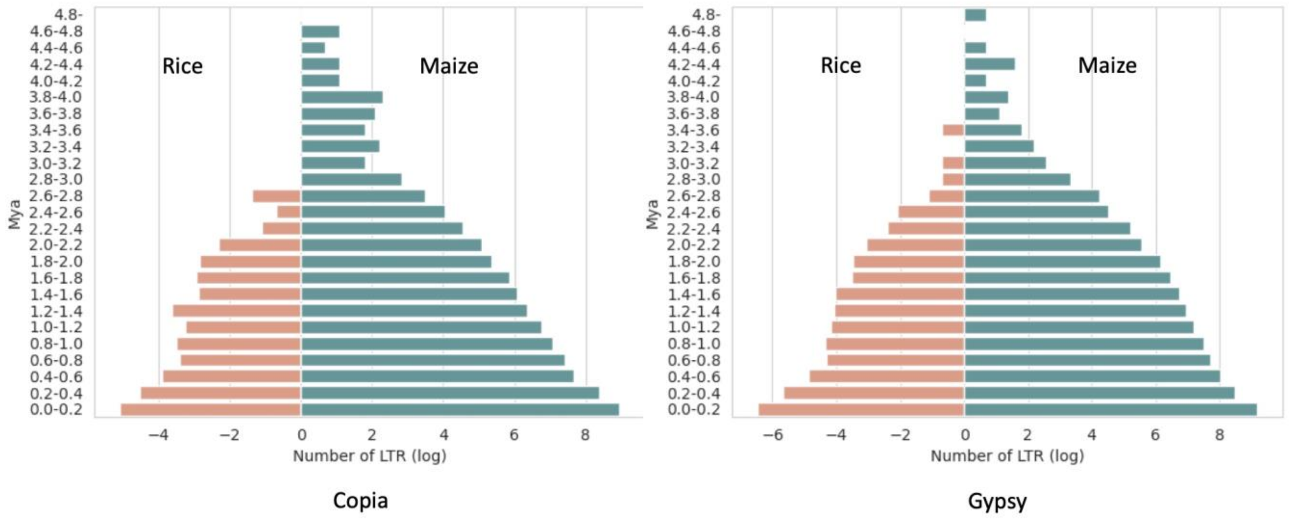

Supplementary Fig. 12 Distributions of insertion times of LTR-RTs in rice and maize genomes calculated using mutation rates of 1.3×10^{-8} and 3.3×10^{-8} (per base per year). The x-axis coordinates in the figure represent the logarithmic scale of LTR numbers and the y-axis represents the insertion time of LTRs. Mya: million years ago.

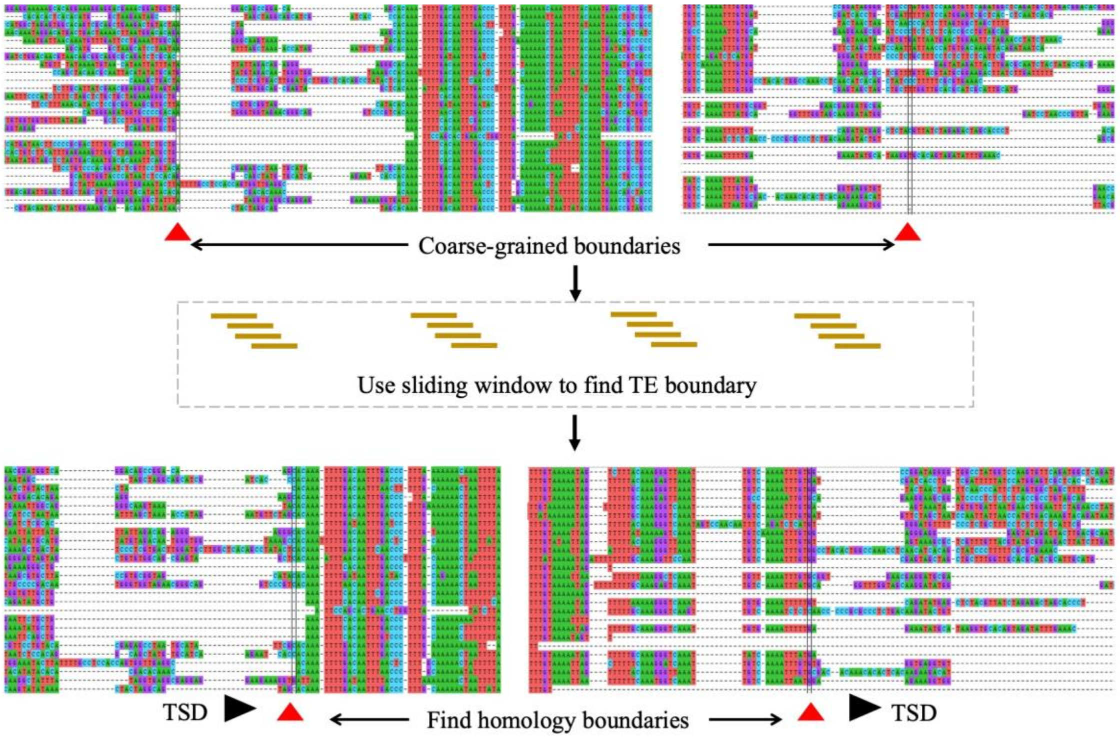

Supplementary Fig. 13 An example of using HiTE to identify homologous boundaries for TIR elements. A sliding window is used to refine coarse-grained boundaries of candidate TIR elements. The red triangles indicate the boundaries, while the black triangles represent TSDs.

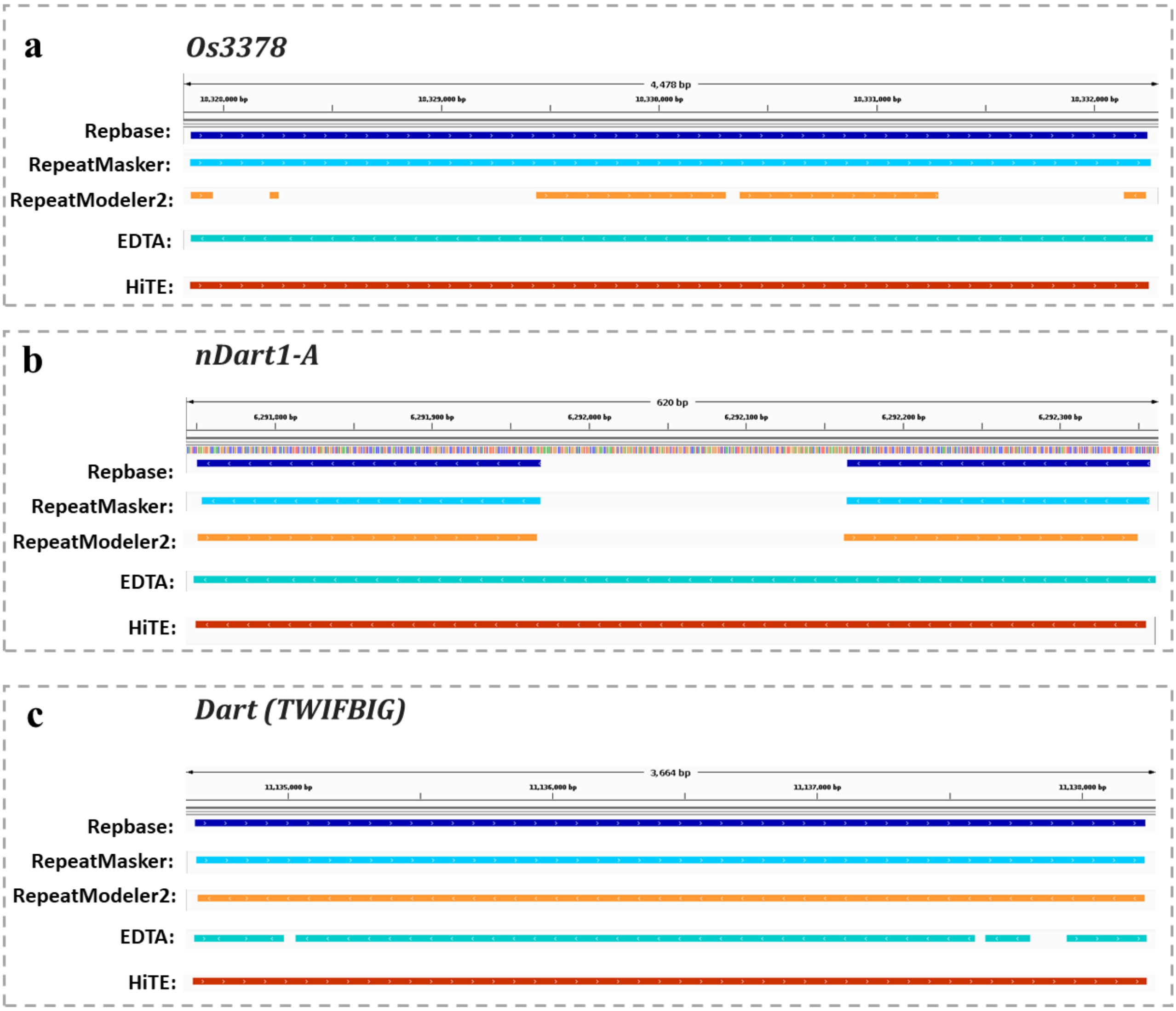

Supplementary Fig. 14 Using different TE libraries for the annotation of known TIR transposons. **a** Repbase, RepeatMasker, and HiTE can accurately annotate the *Os3378* transposon. RepeatModeler2 can only annotate fragmented sequences. EDTA identified a 16,022-bp Helitron element that includes *Os3378*. **b** Only HiTE can annotate the MITE, *nDart*. Interestingly, Repbase and RepeatMasker annotated two terminal sequences of the transposon *TWIFBIG*, indicating that *TWIFBIG* transposon is likely the autonomous element of *nDart*. RepeatModeler2 annotated fragmented sequences of two different TE sequences. EDTA identified a 1,641-bp DNA transposon that includes *nDart*. **c** Except for EDTA, which found fragmented sequences, all other tools were able to annotate the full-length *Dart* (*TWIFBIG*) sequence.

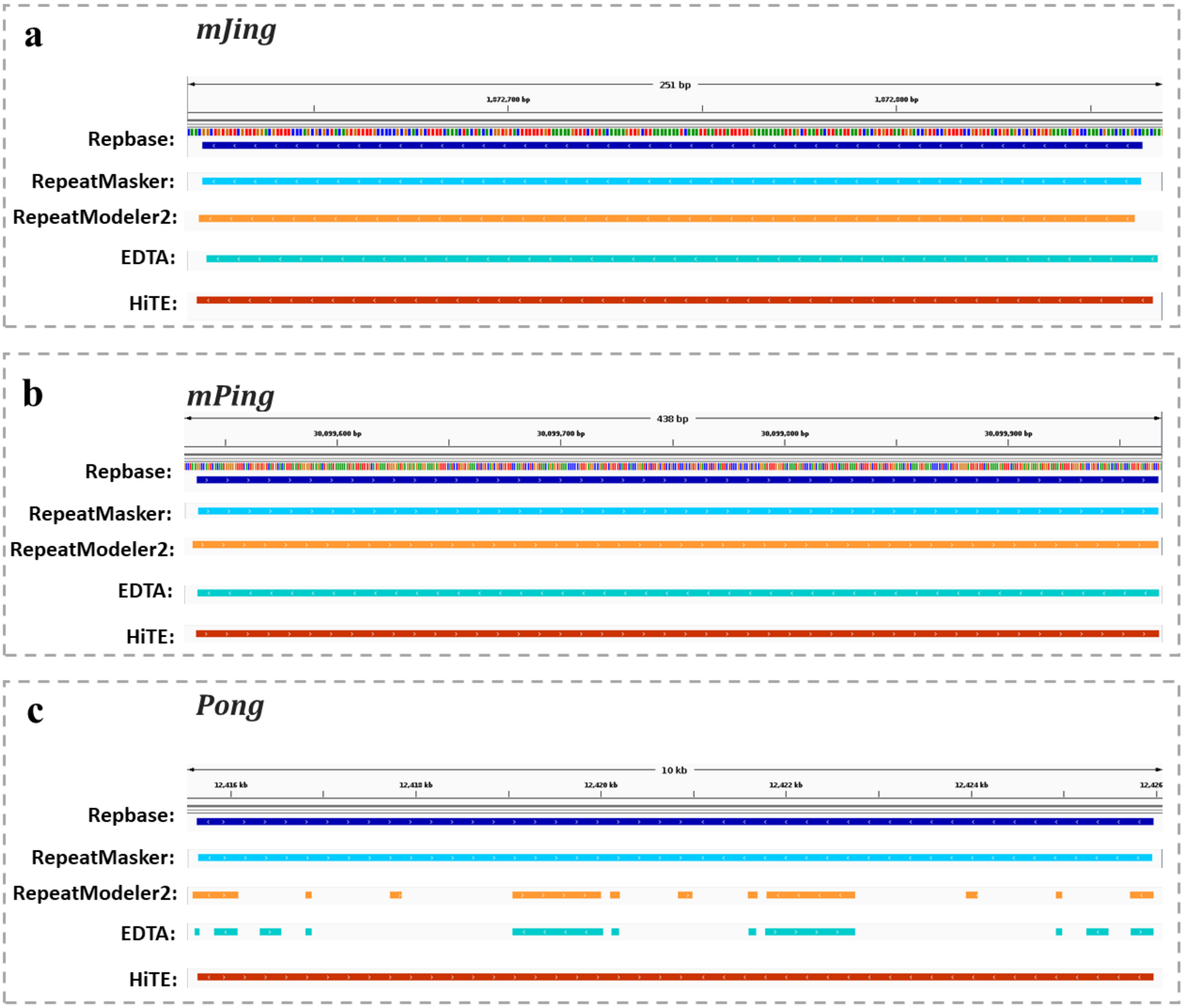

Supplementary Fig. 15 Using different TE libraries for the annotation of known TIR transposons. **a** HiTE identified a 243-bp Harbinger sequence (*mJing*) consistent with Repbase and RepeatMasker, while RepeatModeler2 identified a 245-bp sequence with inaccurate TE boundaries, requiring manual editing. EDTA identified a 19,111-bp DNA transposon containing *mJing*. **b** All tools annotated *mPing*, but the sequence identified by RepeatModeler2 had an inaccurate 5' end. **c** Only Repbase, RepeatMasker, and HiTE were able to accurately annotate *Pong*, while EDTA and RM2 could only annotate fragmented sequences.

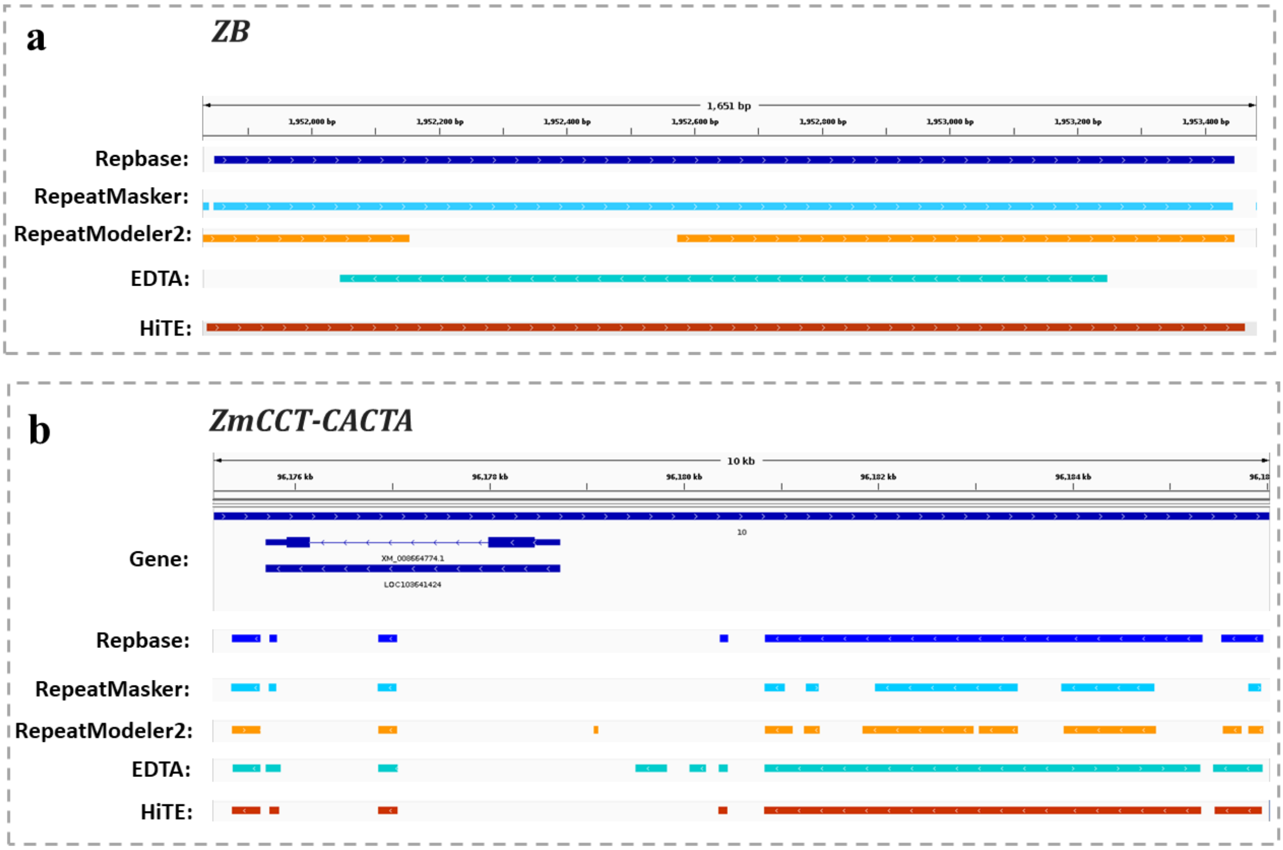

Supplementary Fig. 16 Using different TE libraries for the annotation of known TIR transposons. **a** Only Repbase, RepeatMasker, and HiTE were able to accurately annotate *ZB*, while EDTA and RM2 could only annotate fragmented sequences. **b** A CACTA-like element within the *ZmCCT* promoter, which significantly reduces flowering time, was detected by Repbase and HiTE, while RepeatMasker and RM2 could only annotate fragmented sequences. Although EDTA appears to annotate the element, it annotates fragments of two different TEs.

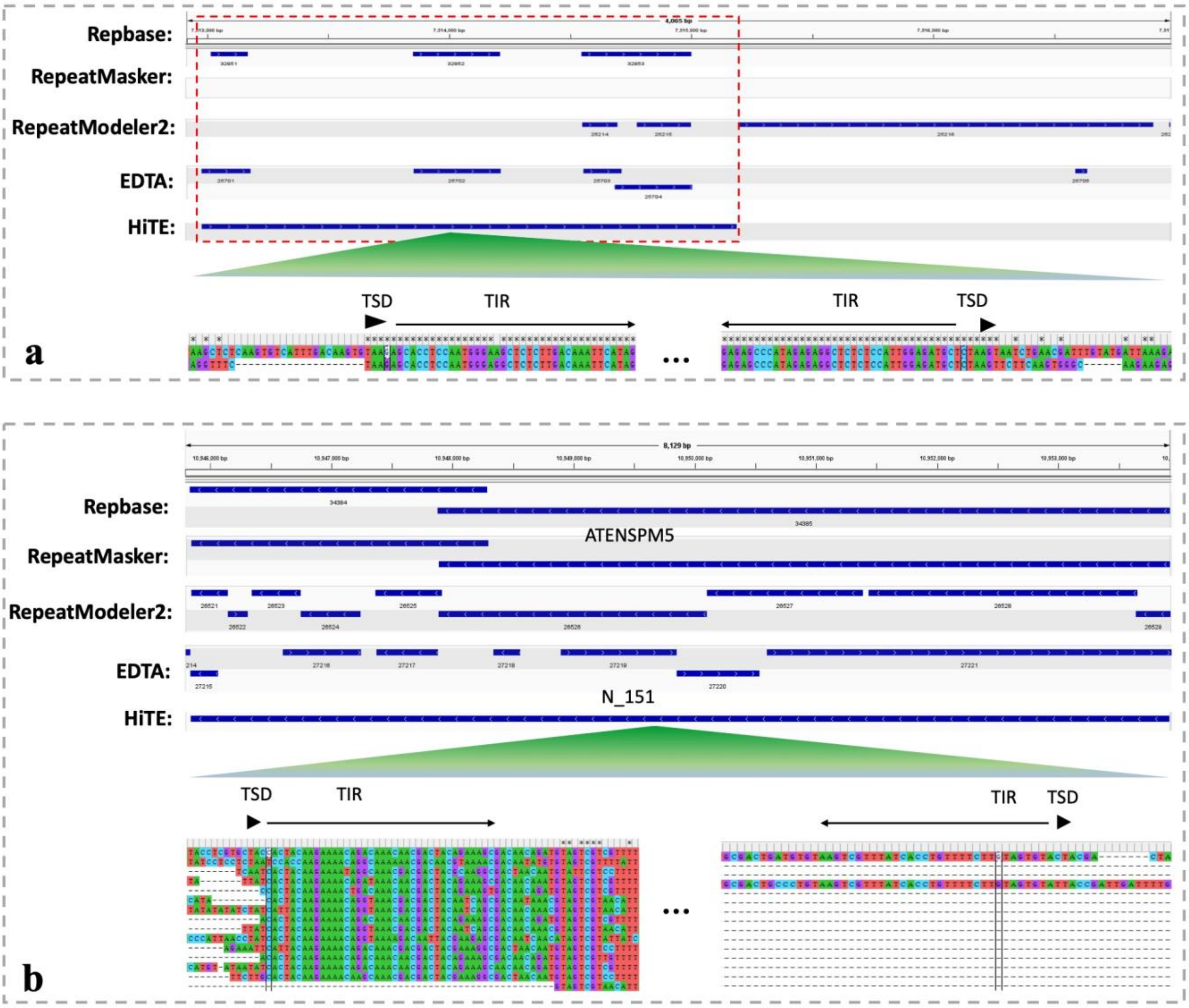

Supplementary Fig. 17 A case study demonstrating the unique TE families in HiTE based on *A. thaliana*. **a** HiTE identified a novel TIR transposon, which possesses two full-length copies, and exhibits clear terminal inverted repeats (TIRs) and target site duplications (TSDs). The target region is highlighted within the red box, while outside the box, RepeatModeler2 annotated a novel L1 element that lacks copies within the genome. **b** HiTE discovered a novel TIR transposon, which consists of two full-length copies as well as multiple truncated copies. In both instances, EDTA and RepeatModeler2 only annotated fragmented TE sequences.

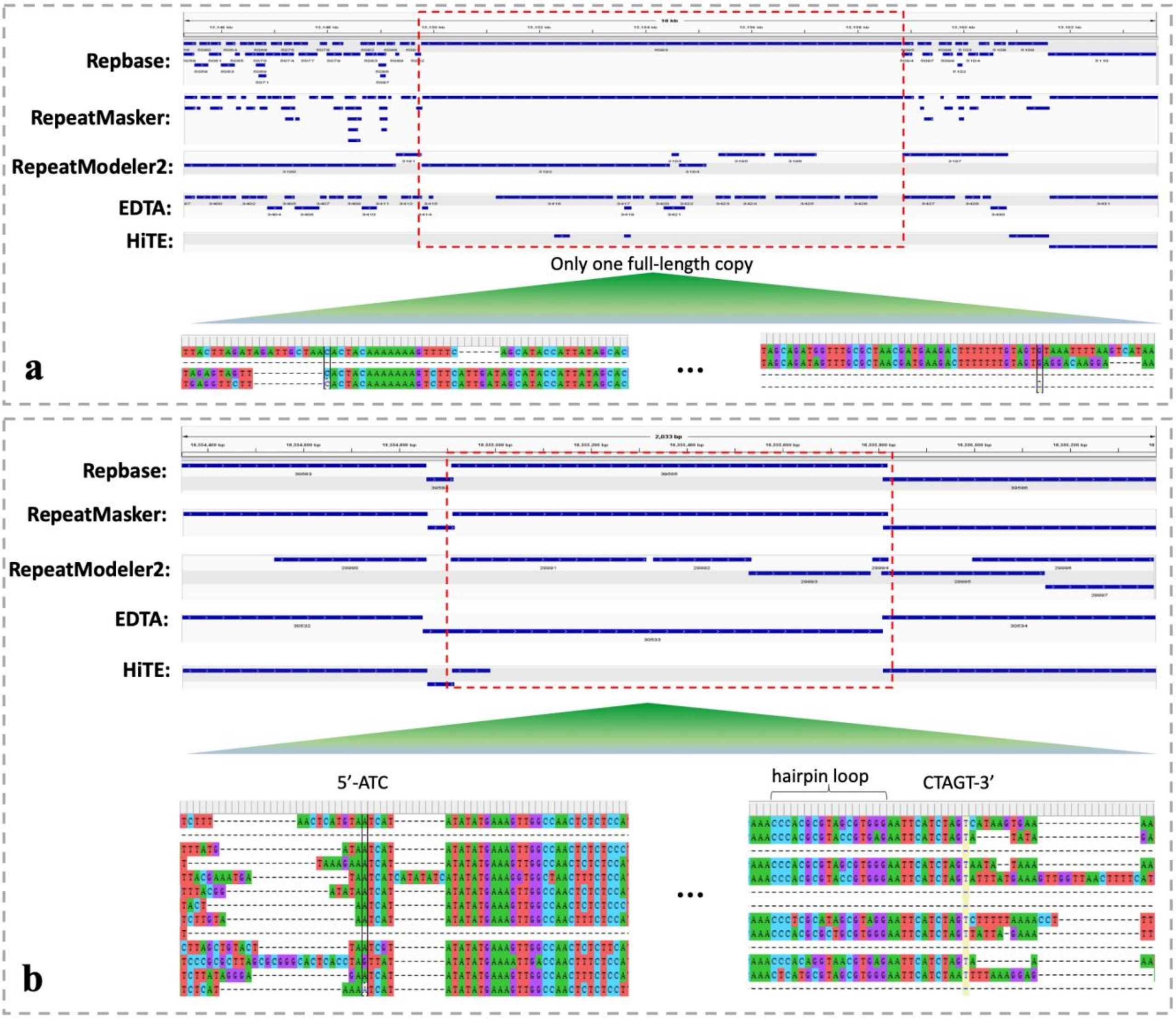

Supplementary Fig. 18 A case study demonstrating the missing TE families in HiTE based on *A. thaliana*. **a** HiTE missed a TIR transposon with only one full-length copy and three truncated copies. Both RepeatModeler2 and EDTA only annotated fragmented TE sequences. The target region is highlighted within the red box, and on either side of the box, Repbase and RepeatMasker annotate it as a satellite, while RepeatModeler2 and EDTA annotate it as fragmented TE sequences. **b** HiTE missed a Helitron transposon, which exhibits a clear 5'-ATC...CTAGT-3' and hairpin loop structure. RepeatModeler2 annotated fragmented TE sequences, and the 5' end recognized by EDTA deviated from the actual boundaries.

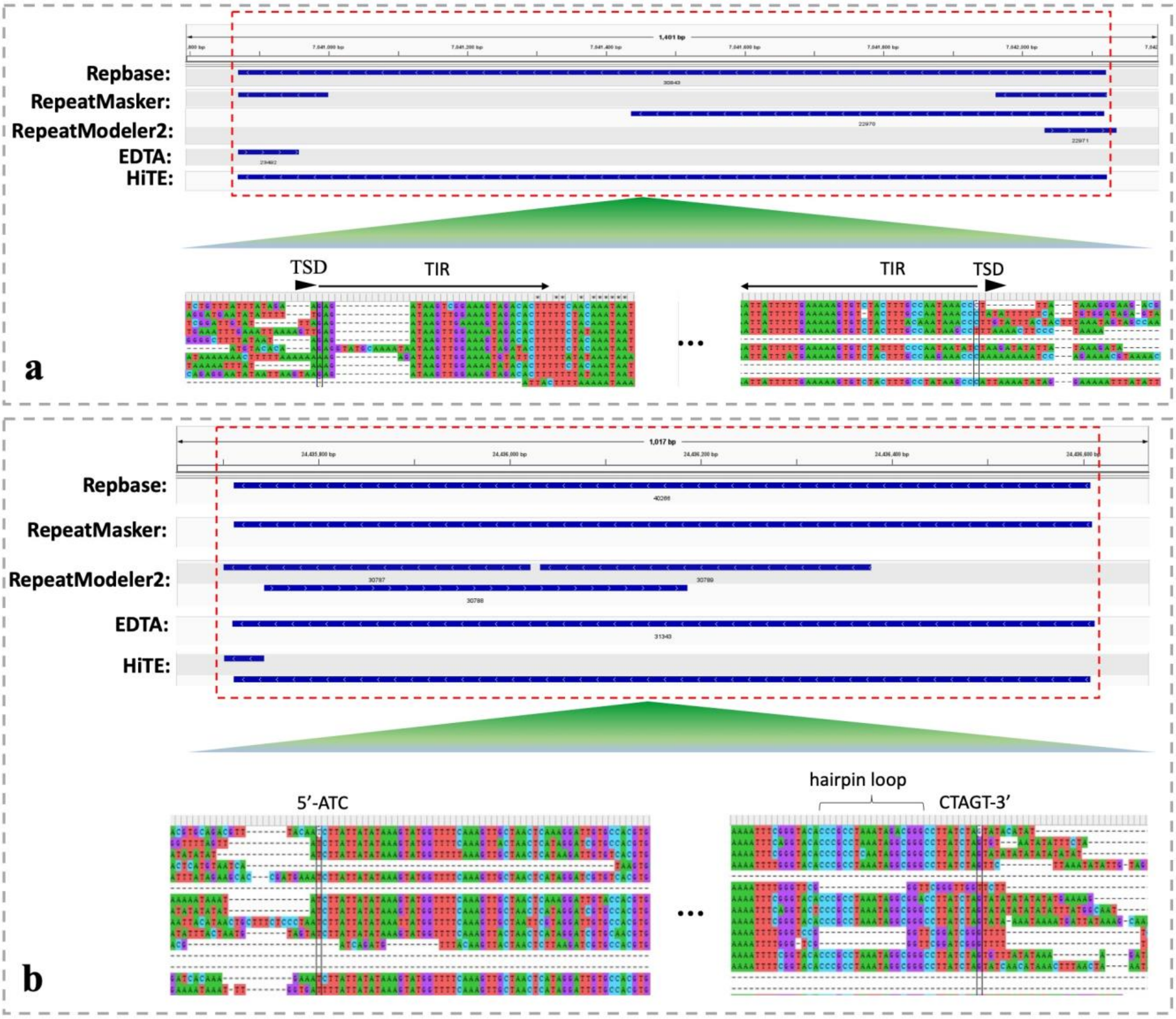

Supplementary Fig. 19 A case study demonstrating the shared TE families between HiTE and other libraries based on *A. thaliana*. **a** HiTE identified a TIR transposon that is shared with Repbase, while the remaining libraries annotated only fragmented TE sequences. The target region is highlighted within the red box. The shared TE sequence exhibits clear terminal inverted repeats (TIRs) and target site duplications (TSDs). **b** HiTE identified a Helitron transposon that is shared with Repbase and RepeatMasker, which exhibits a clear 5'-ATC...CTAGT-3' and hairpin loop structure. RepeatModeler2 annotated fragmented TE sequences, and the sequence annotated by EDTA only represents a small portion of the sequences it recognized.

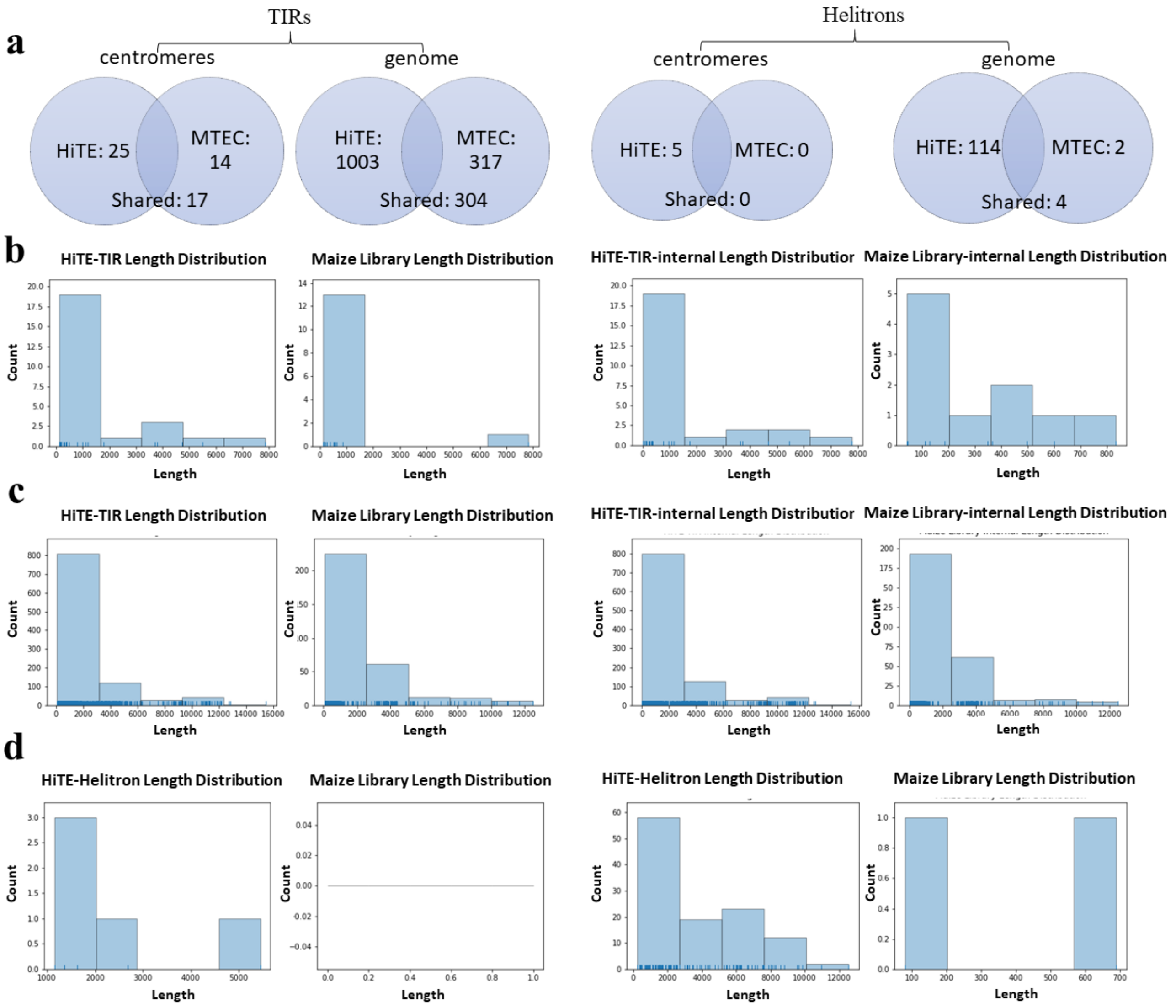

Supplementary Fig. 20 A comparative analysis between HiTE and the Maize TE Consortium (MTEC) library on TIR and Helitron transposons in centromeres and the whole genome. **a** Quantification of the unique and shared TIR (**left**) and Helitron (**right**) transposons in both the centromeres and genome regions of HiTE and MTEC. **b** The length distribution of TIRs (**left**) and TIR internals (**right**) in the centromeres for HiTE and MTEC. **c** The length distribution of TIRs (**left**) and TIR internals (**right**) across the entire genome for HiTE and MTEC. **d** Distribution of the length of Helitrons in the centromeres (**left**) and genome regions (**right**) for HiTE and MTEC.

Supplementary Tables

Supplementary Table 1 TE content in the benchmarking genomes produced by RepeatMasker version 4.1.1 and Repbase version 26.05.

		Class	Size (Mb)	Copy number	Total (%)
O. sativa	LTR	Class I	88.4	46,595	23.61
	Non-LTR	Class I	5.7	13,381	1.51
	TIR	Class II	67.7	230,281	18.09
	Helitron	Class II	17.2	66,469	4.60
	Total	-	179.0	356,726	47.81
C. briggsae	LTR	Class I	0.2	234	0.2
	Non-LTR	Class I	0.9	3,085	0.59
	TIR	Class II	14.5	68,146	13.41
	Helitron	Class II	1.8	8,509	1.63
	Total	-	17.4	79,974	15.83
D. melanogaster	LTR	Class I	19.9	21,050	11.78
	Non-LTR	Class I	10.8	15,428	6.37
	TIR	Class II	2.6	6,204	1.53
	Helitron	Class II	1.0	4,822	0.60
	Total	-	34.2	47,504	20.28
D. rerio	LTR	Class I	119.0	296,556	7.09
	Non-LTR	Class I	74.3	215,393	4.42
	TIR	Class II	719.4	3,372,500	42.84
	Helitron	Class II	50.5	178,913	3.01
	Total	-	963.2	4,063,362	57.36
Z. mays	LTR	Class I	1583.4	1,230,464	72.54
	Non-LTR	Class I	55.1	235,690	2.52
	TIR	Class II	109.1	276,230	5.00
	Helitron	Class II	15.4	62,528	0.71
	Total	-	1763.0	1,804,912	80.77
A. thaliana	LTR	Class I	9.1	12,110	7.59
	Non-LTR	Class I	1.8	3,697	1.51
	TIR	Class II	6.3	13,820	5.25
	Helitron	Class II	2.7	8,056	2.29
	Total	-	19.9	37,683	16.64
G. gallus	LTR	Class I	13.8	32,141	1.29
	Non-LTR	Class I	55.1	144,692	5.18
	TIR	Class II	7.7	19,032	0.72
	Helitron	Class II	0	0	0
	Total	-	76.6	195,865	7.19
T. guttata	LTR	Class I	20.1	44,311	1.83
	Non-LTR	Class I	0.1	134	0.01
	TIR	Class II	0	0	0
	Helitron	Class II	0	0	0
	Total	-	20.2	44,445	1.84
M. musculus	LTR	Class I	218.9	723,371	8.02
	Non-LTR	Class I	538.0	1,220,423	19.73
	TIR	Class II	6.0	33,834	0.22
	Helitron	Class II	0	0	0
	Total	-	762.9	1,977,628	27.97

Supplementary Table 2 Performance evaluation of general-purpose TE annotators using different genomes based on the benchmarking method of RepeatModeler2 (BM_RM2). Since RepeatScout cannot process genomes larger than 1 GB, its results are missing in *D. rerio*, *Z. mays*, *G. gallus*, *T. guttata*, and M. musculus. Due to the extensive output size of HiTE-FMEA, its results are missing in *D. rerio*, *Z. mays*, *G. gallus*, *T. guttata*, and *M. musculus* as well. Pe: Perfect; Go: Good; Pr: Present; NF: Not_found; NA: Not applicable.

Species	Tools	BM_RM2 (coverage>0.95)				BM_RM2 (coverage>0.99)			
		Pe	*Go*	*Pr*	*NF*	*Pe*	*Go*	*Pr*	*NF*
C. briggsae	RepeatScout	57	19	55	83	28	48	55	83
	EDTA (sensitive)	11	32	62	109	7	36	62	109
	RepeatModeler2	36	16	61	101	16	36	61	101
	EarlGrey	93	33	25	63	26	100	25	63
	HiTE-FMEA	93	51	60	10	63	81	60	10
	HiTE	**97**	10	22	85	**84**	23	22	85
A. thaliana	RepeatScout	45	44	97	734	23	66	97	734
	EDTA (sensitive)	175	53	110	582	165	63	110	582
	RepeatModeler2	143	51	82	644	128	66	82	644
	EarlGrey	48	82	60	730	16	114	60	730
	HiTE-FMEA	193	110	127	490	129	174	127	490
	HiTE	**282**	23	53	562	**263**	42	53	562
D. melanogaster	RepeatScout	21	102	46	137	12	111	46	137
	EDTA (sensitive)	45	43	38	180	30	58	38	180
	RepeatModeler2	61	42	37	166	53	50	37	166
	EarlGrey	27	110	23	146	19	118	23	146
	HiTE-FMEA	32	109	45	120	18	123	45	120
	HiTE	**78**	31	17	180	**64**	45	17	180
O. sativa	RepeatScout	171	139	359	2,379	76	234	359	2,379
	EDTA (sensitive)	508	271	879	1,390	489	290	879	1,390
	RepeatModeler2	378	104	366	2,200	294	188	366	2,200
	EarlGrey	276	247	284	2,241	117	406	284	2,241
	HiTE-FMEA	899	424	607	1,118	663	660	607	1,118
	HiTE	**1,078**	100	395	1,475	**998**	180	395	1,475
D. rerio	RepeatScout	NA	NA	NA	NA	NA	NA	NA	NA
	EDTA (sensitive)	476	254	713	839	430	300	713	839
	RepeatModeler2	483	147	290	1,362	389	241	290	1,362
	EarlGrey	509	331	247	1,195	181	659	247	1,195

	HiTE-FMEA	NA	NA	NA	NA	NA	NA	NA	NA
	HiTE	**1,120**	139	237	786	**1,025**	234	237	786
Z. mays	RepeatScout	NA	NA	NA	NA	NA	NA	NA	NA
	EDTA (sensitive)	303	115	396	347	269	149	396	347
	RepeatModeler2	341	86	233	501	299	128	233	501
	EarlGrey	48	172	101	840	18	202	101	840
	HiTE-FMEA	NA	NA	NA	NA	NA	NA	NA	NA
	HiTE	**446**	82	335	298	**396**	132	335	298
G. gallus	RepeatScout	NA	NA	NA	NA	NA	NA	NA	NA
	EDTA (sensitive)	10	5	9	36	7	8	9	36
	RepeatModeler2	10	3	11	36	8	5	11	36
	EarlGrey	6	15	8	31	2	19	8	31
	HiTE-FMEA	NA	NA	NA	NA	NA	NA	NA	NA
	HiTE	**15**	3	8	34	**11**	7	8	34
T. guttata	RepeatScout	NA	NA	NA	NA	NA	NA	NA	NA
	EDTA (sensitive)	8	3	11	42	7	4	11	42
	RepeatModeler2	**17**	3	14	30	**10**	10	14	30
	EarlGrey	8	14	12	30	1	21	12	30
	HiTE-FMEA	NA	NA	NA	NA	NA	NA	NA	NA
	HiTE	11	2	10	41	**10**	3	10	41
M. musculus	RepeatScout	NA	NA	NA	NA	NA	NA	NA	NA
	EDTA (sensitive)	34	12	173	338	31	14	174	338
	RepeatModeler2	45	17	110	385	24	38	110	385
	EarlGrey	63	50	133	311	24	89	133	311
	HiTE-FMEA	NA	NA	NA	NA	NA	NA	NA	NA
	HiTE	**86**	20	125	326	**64**	42	125	326

Supplementary Table 3 Performance evaluation of general-purpose TE annotators using different genomes based on the benchmarking method of EDTA (BM_EDTA). Since RepeatScout cannot process genomes larger than 1 GB, its results are missing in *D. rerio*, *Z. mays*, *G. gallus*, *T. guttata*, and *M. musculus*. Due to the extensive output size of HiTE-FMEA, its results are missing in *D. rerio*, *Z. mays*, *G. gallus*, *T. guttata*, and *M. musculus* as well. Sen: Sensitivity; Spe: Specificity; Acc: Accuracy; Pre: Precision; F1: F1-Score; NA: Not applicable.

Species	Tools	BM_EDTA				
		Sen	*Spe*	*Acc*	*Pre*	*F1*
C. briggsae	RepeatScout	0.9668	0.8793	0.8947	0.6308	0.7634
	EDTA (sensitive)	0.9512	0.8345	0.8552	0.5526	0.6991
	RepeatModeler2	0.9567	0.8799	0.8934	0.6308	0.7603
	EarlGrey	0.9735	0.8750	0.8922	0.6231	0.7598
	HiTE-FMEA	**0.9936**	0.7004	0.7511	0.4095	0.5800
	HiTE	0.8986	0.9429	0.9349	**0.7777**	**0.8337**
A. thaliana	RepeatScout	0.8214	0.9639	0.9378	0.8368	0.8290
	EDTA (sensitive)	0.8251	0.9578	0.9349	0.8026	0.8137
	RepeatModeler2	0.8029	0.9655	0.9372	0.8305	0.8165
	EarlGrey	0.8056	0.9589	0.9323	0.8046	0.8051
	HiTE-FMEA	**0.8822**	0.7497	0.7721	0.4172	0.5665
	HiTE	0.7519	0.9894	0.9473	**0.9386**	**0.8349**
D. melanogaster	RepeatScout	**0.9798**	0.8997	0.9176	0.7373	0.8414
	EDTA (sensitive)	0.9385	0.9052	0.9127	0.7448	0.8305
	RepeatModeler2	0.9644	0.9310	0.9385	0.8028	**0.8762**
	EarlGrey	0.3942	0.7319	0.6442	0.3401	0.3652
	HiTE-FMEA	0.9404	0.8658	0.8826	0.6717	0.7837
	HiTE	0.7982	0.9904	0.9435	**0.9641**	0.8733
O. sativa	RepeatScout	0.8930	0.9422	0.9181	0.9370	0.9145
	EDTA (sensitive)	**0.9524**	0.8364	0.8927	0.8460	0.8961
	RepeatModeler2	0.8953	0.9263	0.9111	0.9212	0.9081
	EarlGrey	0.9106	0.9292	0.9201	0.9248	0.9177
	HiTE-FMEA	0.9406	0.6761	0.8049	0.7336	0.8243
	HiTE	0.9105	0.9593	0.9354	**0.9554**	**0.9324**
D. rerio	RepeatScout	NA	NA	NA	NA	NA
	EDTA (sensitive)	0.9484	0.8572	0.9103	0.9026	0.9249
	RepeatModeler2	0.9266	0.9051	0.9177	0.9318	0.9292

	EarlGrey	**0.9592**	0.8736	0.9234	0.9134	0.9357
	HiTE-FMEA	NA	NA	NA	NA	NA
	HiTE	0.9100	0.9535	0.9281	**0.9649**	**0.9367**
Z. mays	RepeatScout	NA	NA	NA	NA	NA
	EDTA (sensitive)	**0.9913**	0.5060	0.8889	0.8824	0.9337
	RepeatModeler2	0.9827	0.7035	0.9240	0.9256	**0.9533**
	EarlGrey	0.9814	0.7069	0.9237	**0.9264**	0.9531
	HiTE-FMEA	NA	NA	NA	NA	NA
	HiTE	0.9882	0.6550	0.9180	0.9148	0.9501
G. gallus	RepeatScout	NA	NA	NA	NA	NA
	EDTA (sensitive)	**0.9810**	0.9387	0.9422	0.5920	0.7384
	RepeatModeler2	0.9784	0.9526	0.9548	0.6522	0.7827
	EarlGrey	0.2914	0.8666	0.8068	0.2024	0.2389
	HiTE-FMEA	NA	NA	NA	NA	NA
	HiTE	0.8664	0.9798	0.9700	**0.8021**	**0.8330**
T. guttata	RepeatScout	NA	NA	NA	NA	NA
	EDTA (sensitive)	0.4784	0.8978	0.8978	0.0008	0.0016
	RepeatModeler2	0.9713	0.9125	0.9125	0.0018	0.0035
	EarlGrey	**0.9718**	0.9213	0.9221	0.1729	0.2935
	HiTE-FMEA	NA	NA	NA	NA	NA
	HiTE	0.9209	0.9566	0.9560	**0.2682**	**0.4155**
M. musculus	RepeatScout	NA	NA	NA	NA	NA
	EDTA (sensitive)	0.9579	0.8286	0.8673	0.7046	0.8119
	RepeatModeler2	0.9550	0.8507	0.8820	0.7324	0.8290
	EarlGrey	**0.9802**	0.8168	0.8652	0.6925	0.8116
	HiTE-FMEA	NA	NA	NA	NA	NA
	HiTE	0.8925	0.9107	0.9051	**0.8162**	**0.8527**

Supplementary Table 4 Performance evaluation of general-purpose TE annotators using different genomes based on the benchmarking method of HiTE (BM_HiTE). Since RepeatScout cannot process genomes larger than 1 GB, its results are missing in *D. rerio*, *Z. mays*, *G. gallus*, *T. guttata*, and *M. musculus*. Due to the extensive output size of HiTE-FMEA, its results are missing in *D. rerio*, *Z. mays*, *G. gallus*, *T. guttata*, and *M. musculus* as well. Sen: Sensitivity; Pre: Precision; F1: F1-Score; NA: Not applicable.

Species	Tools	BM_HiTE (coverage>0.8)			BM_HiTE (coverage>0.95)			BM_HiTE (coverage>0.99)		
		Sen	*Pre*	*F1*	*Sen*	*Pre*	*F1*	*Sen*	*Pre*	*F1*
C. briggsae	RepeatScout	0.7083	0.2043	0.3172	0.6188	0.2222	0.3270	0.4255	0.1711	0.2440
	EDTA (sensitive)	0.1981	0.1923	0.1952	0.0800	0.0888	0.0841	0.0576	0.0642	0.0607
	RepeatModeler2	0.6519	0.3252	0.4339	0.4755	0.2438	0.3223	0.3126	0.1977	0.2422
	EarlGrey	0.9039	0.5451	0.6801	0.8448	**0.6454**	**0.7318**	0.5089	0.5101	0.5095
	HiTE-FMEA	**0.9970**	0.2787	0.4356	**0.9892**	0.1835	0.3095	**0.9435**	0.077	0.1424
	HiTE	0.8391	**0.6378**	**0.7247**	0.8079	0.6407	0.7147	0.7455	**0.5931**	**0.6606**
A. thaliana	RepeatScout	0.2575	0.1664	0.2022	0.0906	0.0720	0.0802	0.0808	0.0668	0.0731
	EDTA (sensitive)	0.8191	0.8981	0.8568	0.8303	0.8970	0.8624	0.8433	0.8933	0.8676
	RepeatModeler2	0.4116	0.4611	0.4349	0.1361	0.1988	0.1616	0.1144	0.1891	0.1425
	EarlGrey	0.6542	0.4615	0.5412	0.2346	0.1903	0.2101	0.1159	0.1059	0.1107
	HiTE-FMEA	**0.9902**	0.4601	0.6283	0.9100	0.1376	0.2391	0.7344	0.0714	0.1301
	HiTE	0.9016	**0.9589**	**0.9294**	**0.9125**	**0.9582**	**0.9348**	**0.9169**	**0.9547**	**0.9354**
D. melanogaster	RepeatScout	0.5465	0.0820	0.1426	0.2112	0.0358	0.0613	0.2068	0.0426	0.0707
	EDTA (sensitive)	0.3673	0.2165	0.2724	0.2921	0.1918	0.2316	0.2539	0.1735	0.2061
	RepeatModeler2	0.7252	0.2174	0.3346	0.6775	0.2336	0.3475	0.6294	0.2191	0.3251
	EarlGrey	0.8788	0.4684	0.6111	0.5263	0.1700	0.2569	0.3864	0.1213	0.1846
	HiTE-FMEA	**0.9981**	0.0708	0.1323	**0.9612**	0.0276	0.0536	**0.8870**	0.0156	0.0307
	HiTE	0.8041	**0.6904**	**0.7429**	0.7130	**0.5592**	**0.6268**	0.7072	**0.5388**	**0.6116**
O. sativa	RepeatScout	0.6460	0.4416	0.5246	0.3938	0.3038	0.3430	0.3277	0.2908	0.3082
	EDTA (sensitive)	0.9363	0.8968	0.9161	0.8873	0.8599	0.8734	0.8908	0.8555	0.8728
	RepeatModeler2	0.7381	0.7099	0.7237	0.5315	0.5661	0.5482	0.4729	0.5279	0.4989
	EarlGrey	0.8300	0.7036	0.7616	0.6492	0.5041	0.5675	0.5543	0.4598	0.5026
	HiTE-FMEA	**0.9998**	0.6826	0.8113	**0.9973**	0.4273	0.5983	**0.9908**	0.3119	0.4744
	HiTE	0.9876	**0.9182**	**0.9516**	0.9800	**0.8951**	**0.9356**	0.9737	**0.8816**	**0.9253**
D. rerio	RepeatScout	NA	NA	NA	NA	NA	NA	NA	NA	NA
	EDTA (sensitive)	0.7491	**0.7838**	0.7661	0.6374	0.7720	0.6983	0.6026	**0.7727**	0.6771
	RepeatModeler2	0.6444	0.6803	0.6619	0.5243	0.5728	0.5475	0.3900	0.4573	0.4210
	EarlGrey	0.9123	0.7483	0.8222	0.9067	**0.8239**	**0.8633**	0.6982	0.7466	0.7216

	HiTE-FMEA	NA	NA	NA	NA	NA	NA	NA	NA	NA
	HiTE	**0.9670**	0.7769	**0.8616**	**0.9713**	0.7671	0.8572	**0.9564**	0.7072	**0.8131**
Z. mays	RepeatScout	NA	NA	NA	NA	NA	NA	NA	NA	NA
	EDTA (sensitive)	0.9370	0.7625	0.8408	0.8865	0.7477	0.8112	0.8802	0.7968	0.8364
	RepeatModeler2	0.7321	0.6439	0.6852	0.4658	0.4250	0.4445	0.1970	0.1828	0.1896
	EarlGrey	0.9510	0.6683	0.7850	0.1663	0.0653	0.0938	0.1204	0.0717	0.0899
	HiTE-FMEA	NA	NA	NA	NA	NA	NA	NA	NA	NA
	HiTE	**0.9712**	**0.8244**	**0.8918**	**0.9685**	**0.8314**	**0.8947**	**0.9685**	**0.8429**	**0.9013**
G. gallus	RepeatScout	NA	NA	NA	NA	NA	NA	NA	NA	NA
	EDTA (sensitive)	0.3883	0.1113	0.1730	0.4373	0.1055	0.1700	0.5035	0.1009	0.1681
	RepeatModeler2	0.5264	0.1184	0.1933	0.5421	0.1319	0.2121	0.5475	0.1290	0.2089
	EarlGrey	0.7876	0.0538	0.1008	0.6228	0.0340	0.0645	0.5077	0.0240	0.0459
	HiTE-FMEA	NA	NA	NA	NA	NA	NA	NA	NA	NA
	HiTE	**0.8577**	**0.2366**	**0.3709**	**0.8833**	**0.2701**	**0.4137**	**0.8869**	**0.2986**	**0.4467**
T. guttata	RepeatScout	NA	NA	NA	NA	NA	NA	NA	NA	NA
	EDTA (sensitive)	0.5472	0.2837	0.3737	0.4823	0.2568	0.3351	0.3784	0.2090	0.2693
	RepeatModeler2	**0.8550**	0.3760	0.5223	0.6522	0.2941	0.4054	0.5219	0.2451	0.3335
	EarlGrey	0.8247	0.2722	0.4093	**0.7483**	0.2393	0.3627	**0.6260**	0.1886	0.2898
	HiTE-FMEA	NA	NA	NA	NA	NA	NA	NA	NA	NA
	HiTE	0.7283	**0.4768**	**0.5763**	0.6512	**0.4811**	**0.5534**	0.5981	**0.4984**	**0.5437**
M. musculus	RepeatScout	NA	NA	NA	NA	NA	NA	NA	NA	NA
	EDTA (sensitive)	0.0527	0.4047	0.0932	0.1152	0.2602	0.1597	0.0802	0.1541	0.1055
	RepeatModeler2	0.9586	0.9695	0.9641	0.5396	0.5270	0.5332	0.2857	0.3575	0.3176
	EarlGrey	0.9962	0.9092	0.9507	0.9807	**0.8232**	**0.8951**	**0.8629**	**0.6743**	**0.7570**
	HiTE-FMEA	NA	NA	NA	NA	NA	NA	NA	NA	NA
	HiTE	**0.9998**	**0.9882**	**0.9940**	**0.9855**	0.4605	0.6277	0.8460	0.1292	0.2242

Supplementary Table 5 Performance evaluation of different tools across various TE types using *O. sativa* and *A. thaliana* genomes based on the benchmarking method of RepeatModeler2 (BM_RM2).

Species	TE	Tools	BM_RM2 (coverage>0.95)			
			Perfect	*Good*	*Present*	*Not_found*
O. sativa	TIR	EDTA-NoFiltering	208	106	326	834
		EDTA	165	112	320	877
		HiTE-NoFiltering	**613**	112	363	386
		HiTE	476	26	165	807
		RepeatModeler2	79	27	199	1,169
	Helitron	EDTA-NoFiltering	3	66	77	164
		EDTA	4	30	54	222
		HiTE-NoFiltering	**104**	25	63	118
		HiTE	71	10	21	208
		RepeatModeler2	8	7	27	268
A. thaliana	TIR	EDTA-NoFiltering	17	8	11	169
		EDTA	14	8	8	175
		HiTE-NoFiltering	**77**	18	41	69
		HiTE	49	3	8	145
		RepeatModeler2	8	8	14	175
	Helitron	EDTA-NoFiltering	13	16	10	9
		EDTA	9	14	9	16
		HiTE-NoFiltering	**20**	6	11	11
		HiTE	19	1	6	22
		RepeatModeler2	2	2	5	39

Supplementary Table 6 Performance evaluation of different tools across various TE types using *O. sativa* and *A. thaliana* genomes based on the benchmarking method of EDTA (BM_EDTA). Sen: Sensitivity; Spe: Specificity; Acc: Accuracy; Pre: Precision; F1: F1-Score.

Species	TE	Tools	BM_EDTA				
			Sen	*Spe*	*Acc*	*Pre*	*F1*
O. sativa	TIR	EDTA-NoFiltering	0.8870	0.6292	0.6813	0.3771	0.5292
		EDTA	0.8274	0.6989	0.7253	0.4149	0.5526
		HiTE-NoFiltering	**0.9636**	0.5792	0.6553	0.3609	0.5251
		HiTE	0.8563	0.9827	0.9570	**0.9267**	**0.8901**
		RepeatModeler2	0.8022	0.9634	0.9301	0.8508	0.8258
	Helitron	EDTA-NoFiltering	**0.9575**	0.4991	0.5412	0.1622	0.2773
		EDTA	0.8930	0.6259	0.6509	0.1982	0.3244
		HiTE-NoFiltering	0.9528	0.5633	0.5992	0.1811	0.3044
		HiTE	0.7397	0.9765	0.9532	0.7743	**0.7566**
		RepeatModeler2	0.6633	0.9899	0.9570	**0.8804**	**0.7566**
A. thaliana	TIR	EDTA-NoFiltering	0.5263	0.9286	0.9023	0.3406	0.4136
		EDTA	0.5027	0.9492	0.9196	0.4127	0.4533
		HiTE-NoFiltering	**0.8862**	0.8419	0.8443	0.2465	0.3858
		HiTE	0.5096	0.9963	0.9641	0.9066	0.6525
		RepeatModeler2	0.6308	0.9958	0.9732	**0.9076**	**0.7443**
	Helitron	EDTA-NoFiltering	**0.9664**	0.7833	0.7876	0.0958	0.1744
		EDTA	0.8692	0.8937	0.8931	0.1687	0.2826
		HiTE-NoFiltering	0.9122	0.9105	0.9105	0.1986	0.3262
		HiTE	0.6419	0.9961	0.9866	0.8200	0.7201
		RepeatModeler2	0.6425	0.9972	0.9877	**0.8659**	**0.7377**

Supplementary Table 7 Performance evaluation of different tools across various TE types using *O. sativa* and *A. thaliana* genomes based on the benchmarking method of HiTE (BM_HiTE). Sen: Sensitivity; Pre: Precision; F1: F1-Score.

Species	TE	Tools	BM_HiTE (coverage>0.95)		
			Sen	*Pre*	*F1*
O. sativa	TIR	EDTA-NoFiltering	0.9492	0.5679	0.7106
		EDTA	0.9385	0.6897	0.7951
		HiTE-NoFiltering	**0.9974**	0.6487	0.7861
		HiTE	0.9737	**0.9058**	**0.9385**
		RepeatModeler2	0.1164	0.1629	0.1358
	Helitron	EDTA-NoFiltering	0.9764	0.3976	0.5651
		EDTA	0.9490	0.3836	0.5464
		HiTE-NoFiltering	**0.9892**	0.0306	0.0594
		HiTE	0.8749	**0.7381**	**0.8007**
		RepeatModeler2	0.0750	0.1484	0.0996
A. thaliana	TIR	EDTA-NoFiltering	0.2699	0.036	0.0636
		EDTA	0.2328	0.3806	0.2889
		HiTE-NoFiltering	**0.7891**	0.2925	0.4268
		HiTE	0.4193	**0.4470**	**0.4327**
		RepeatModeler2	0.0235	0.0615	0.0340
	Helitron	EDTA-NoFiltering	0.9215	0.5832	0.7143
		EDTA	0.8528	0.6290	0.7240
		HiTE-NoFiltering	**0.9914**	0.3837	0.5532
		HiTE	0.8214	**0.9026**	**0.8601**
		RepeatModeler2	0.058	0.1885	0.0887

Supplementary Table 8 Performance evaluation of the filtering methods in HiTE on TIR transposons using *C. briggsae* genome based on three distinct assessment approaches. Sen: Sensitivity; Spe: Specificity; Acc: Accuracy; Pre: Precision; F1: F1-Score.

Species	Benchmarking methods	Metrics	HiTE-NoFiltering	HiTE
C. briggsae	BM_EDTA	*Sen*	**0.9910**	0.8740
		Spe	0.8282	0.9494
		Acc	0.8532	0.9371
		Pre	0.5115	**0.7714**
		F1	0.6747	**0.8195**
	BM_HiTE (coverage>0.95)	*Sen*	**0.9697**	0.8025
		Pre	0.3083	**0.5916**
		F1	0.4678	**0.6811**
	BM_RM2 (coverage>0.95)	*Perfect*	**75**	72
		Good	22	8
		Present	66	22
		Not_found	15	76

Supplementary Table 9 Performance evaluation of different non-LTR identification methods based on the benchmarking method of RepeatModeler2 (BM_RM2).

Species	Methods	BM_RM2 (coverage>0.95)			
		Perfect	*Good*	*Present*	*Not_found*
O. sativa	RepeatModeler2	2	2	14	174
	EDTA	1	5	20	166
	HiTE-NonLTR	9	0	3	180
	HiTE-NonLTR-homology	**77**	2	16	97
	HiTE	**77**	3	19	93
A. thaliana	RepeatModeler2	1	0	4	155
	EDTA	1	0	5	154
	HiTE-NonLTR	3	1	1	155
	HiTE-NonLTR-homology	**18**	0	3	139
	HiTE	**18**	1	2	139
D. melanogaster	RepeatModeler2	3	4	6	32
	EDTA	3	4	5	33
	HiTE-NonLTR	10	0	1	34
	HiTE-NonLTR-homology	**18**	2	4	21
	HiTE	18	2	4	21
G. gallus	RepeatModeler2	0	0	0	8
	EDTA	0	0	0	8
	HiTE-NonLTR	0	0	0	8
	HiTE-NonLTR-homology	0	0	2	6
	HiTE	0	0	2	6
T. guttata	RepeatModeler2	0	0	1	0
	EDTA	0	0	1	0
	HiTE-NonLTR	0	0	0	1
	HiTE-NonLTR-homology	**1**	0	0	0
	HiTE	**1**	0	0	0
D. rerio	RepeatModeler2	4	3	13	414
	EDTA	3	4	11	416
	HiTE-NonLTR	38	4	7	385
	HiTE-NonLTR-homology	**191**	9	48	186
	HiTE	**191**	10	47	186
M. musculus	RepeatModeler2	2	1	9	48
	EDTA	2	1	10	47
	HiTE-NonLTR	**10**	6	11	33
	HiTE-NonLTR-homology	0	5	27	28
	HiTE	**10**	9	16	25

Supplementary Table 10 Performance evaluation of different non-LTR identification methods based on the benchmarking method of EDTA (BM_EDTA).

Species	Methods	BM_EDTA		
		Sensitivity	*Precision*	*F1-score*
O. sativa	RepeatModeler2	0.6629	**0.7981**	0.7243
	EDTA	0.7196	0.3873	0.5036
	HiTE-NonLTR	0.4824	0.5487	0.5134
	HiTE-NonLTR-homology	0.7548	0.7580	**0.7564**
	HiTE	**0.7602**	0.5666	0.6493
A. thaliana	RepeatModeler2	0.6492	0.6025	0.6250
	EDTA	0.6185	0.5616	0.5886
	HiTE-NonLTR	0.3916	**0.9919**	0.5615
	HiTE-NonLTR-homology	0.6877	0.9779	0.8075
	HiTE	**0.6946**	0.9734	**0.8107**
D. melanogaster	RepeatModeler2	**0.8754**	0.8471	0.8610
	EDTA	0.8723	0.8427	0.8572
	HiTE-NonLTR	0.6090	**0.9953**	0.7556
	HiTE-NonLTR-homology	0.8453	0.9275	**0.8845**
	HiTE	0.8460	0.9251	0.8838
G. gallus	RepeatModeler2	0.9752	**0.7643**	**0.8570**
	EDTA	**0.9826**	0.6694	0.7963
	HiTE-NonLTR	0.2981	0.4650	0.3633
	HiTE-NonLTR-homology	0.2981	0.2692	0.2829
	HiTE	0.2981	0.2675	0.2819
T. guttata	RepeatModeler2	0.9652	0.0023	0.0047
	EDTA	**0.9657**	**0.0024**	**0.0048**
	HiTE-NonLTR	0	0	0
	HiTE-NonLTR-homology	0.0788	0.0002	0.0005
	HiTE	0.0788	0.0002	0.0005
D. rerio	RepeatModeler2	0.7254	0.6627	**0.6927**
	EDTA	0.7209	**0.6630**	0.6908
	HiTE-NonLTR	0.4094	0.4352	0.4219
	HiTE-NonLTR-homology	0.8946	0.5089	0.6487
	HiTE	**0.8950**	0.4486	0.5977
M. musculus	RepeatModeler2	**0.9489**	0.6518	0.7727
	EDTA	0.9450	**0.6798**	**0.7908**
	HiTE-NonLTR	0.3876	0.3695	0.3783
	HiTE-NonLTR-homology	0.3876	0.3663	0.3766
	HiTE	0.3876	0.3391	0.3618

Supplementary Table 11 Performance evaluation of different non-LTR identification methods based on the benchmarking method of HiTE (BM_HiTE).

Species	Methods	BM_HiTE (coverage>0.95)		
		Sensitivity	*Precision*	*F1-score*
O. sativa	RepeatModeler2	0.0145	0.0202	0.0168
	EDTA	0.0236	0.0231	0.0234
	HiTE-NonLTR	0.1175	0.0405	0.0602
	HiTE-NonLTR-homology	**0.7286**	**0.8546**	**0.7866**
	HiTE	0.7176	0.2415	0.3613
A. thaliana	RepeatModeler2	0.0196	0.0424	0.0268
	EDTA	0.0159	0.0322	0.0213
	HiTE-NonLTR	0.0628	**0.9098**	0.1175
	HiTE-NonLTR-homology	0.1707	0.7683	0.2793
	HiTE	**0.2128**	0.9003	**0.3442**
D. melanogaster	RepeatModeler2	0.6222	0.3709	0.4648
	EDTA	0.6063	0.2592	0.3631
	HiTE-NonLTR	0.8430	**0.7222**	0.7779
	HiTE-NonLTR-homology	0.8903	0.6992	**0.7833**
	HiTE	**0.9232**	0.6663	0.7740
G. gallus	RepeatModeler2	0	0	0
	EDTA	0	0	0
	HiTE-NonLTR	0	0	0
	HiTE-NonLTR-homology	**0.0616**	**0.0008**	**0.0017**
	HiTE	**0.0616**	**0.0008**	**0.0017**
T. guttata	RepeatModeler2	0.3587	0.0023	0.0046
	EDTA	0	0	0
	HiTE-NonLTR	0	0	0
	HiTE-NonLTR-homology	**1.0**	**0.0045**	**0.0090**
	HiTE	**1.0**	**0.0045**	**0.0090**
D. rerio	RepeatModeler2	0.0934	**0.2468**	0.1356
	EDTA	0.3217	0.1756	**0.2272**
	HiTE-NonLTR	0.1609	0.0822	0.1088
	HiTE-NonLTR-homology	0.9209	0.0623	0.1167
	HiTE	**0.9258**	0.0624	0.1169
M. musculus	RepeatModeler2	0.4583	0.4680	0.4631
	EDTA	0.4635	0.4721	0.4678
	HiTE-NonLTR	0.9924	**0.6535**	**0.7880**
	HiTE-NonLTR-homology	0.2164	0.0099	0.0190
	HiTE	**0.9927**	0.4784	0.6456

Supplementary Table 12 The impact of different assembly qualities on the performance of HiTE. Arab: Arabidopsis; Pe: Perfect; Go: Good; Pr: Present; NF: Not_found; Se: Sensitivity; Pre: Precision; F1: F1-score.

Species	Assembly	BM_RM2 (coverage>0.95)				BM_HiTE (coverage>0.8)			BM_HiTE (coverage>0.95)			BM_HiTE (coverage>0.99)		
		Pe	*Go*	*Pr*	*NF*	*Se*	*Pre*	*F1*	*Se*	*Pre*	*F1*	*Se*	*Pre*	*F1*
Arab	Gaps	281	25	57	557	0.9193	0.9659	0.9420	0.9053	0.9440	0.9242	0.8937	0.9264	0.9098
	T2T	**290**	24	56	550	**0.9394**	**0.9678**	**0.9534**	**0.9476**	**0.9627**	**0.9551**	**0.9508**	**0.9600**	**0.9554**
Rice	Gaps	**1,089**	110	382	1,467	0.9848	0.9264	0.9547	0.9727	0.8711	0.9191	0.9584	0.8152	0.8810
	T2T	1,083	107	389	1,469	**0.9890**	**0.9306**	**0.9589**	**0.9832**	**0.9161**	**0.9485**	**0.9738**	**0.8825**	**0.9259**

Supplementary Table 13 Time and resource consumption. Since RepeatScout cannot process genomes larger than 1 GB, its results are missing in *D. rerio*, *Z. mays*, *G. gallus*, *T. guttata*, and *M. musculus*. NA: Not applicable.

Species	Methods	Versions	Library Size	Running Time (h:m:s)	Maximum mem (kbytes)
C. briggsae	RepeatScout	v1.0.5	847 KB	**00:24:00**	2,279,908
	EDTA (sensitive)	v2.0.1	2.5 MB	2:49:02	2,003,524
	RepeatModeler2	v2.0.1	589 KB	5:00:17	918,964
	EarlGrey	v3.0	645 KB	7:29:45	2,311,392
	HiTE	v3.2	388 KB	00:27:22	**859,592**
A. thaliana	RepeatScout	v1.0.5	1.8 MB	**00:22:33**	1,841,608
	EDTA (sensitive)	v2.0.1	2.9 MB	3:06:51	2,284,448
	RepeatModeler2	v2.0.1	1.3 MB	6:11:02	16,960,476
	EarlGrey	v3.0	1.7 MB	10:23:49	26,553,512
	HiTE	v3.2	1.5 MB	00:25:33	**1,261,432**
D. melanogaster	RepeatScout	v1.0.5	2.1 MB	**00:32:35**	3,237,756
	EDTA (sensitive)	v2.0.1	1.9 MB	4:24:05	1,995,344
	RepeatModeler2	v2.0.1	1.1 MB	4:57:57	15,317,336
	EarlGrey	v3.0	2.2 MB	8:43:12	19,544,052
	HiTE	v3.2	961 KB	00:34:37	**3,534,964**
O. sativa	RepeatScout	v1.0.5	5.6 MB	**1:06:26**	7,448,644
	EDTA (sensitive)	v2.0.1	21 MB	10:13:31	4,214,604
	RepeatModeler2	v2.0.1	4.1 MB	25:50:58	25,993,528
	EarlGrey	v3.0	5.2 MB	42:48:26	30,142,976
	HiTE	v3.2	8.8 MB	02:02:54	**2,496,328**
D. rerio	RepeatScout	v1.0.5	NA	NA	NA
	EDTA (sensitive)	v2.0.1	16 MB	60:13:59	33,576,540
	RepeatModeler2	v2.0.1	3.7 MB	34:30:04	24,279,276
	EarlGrey	v3.0	8.8 MB	91:26:38	35,761,548
	HiTE	v3.2	7.9 MB	**12:06:27**	**25,957,256**
Z. mays	RepeatScout	v1.0.5	NA	NA	NA
	EDTA (sensitive)	v2.0.1	33 MB	110:56:02	21,660,416
	RepeatModeler2	v2.0.1	5.6 MB	**24:10:44**	17,911,656
	EarlGrey	v3.0	6.2 MB	71:54:17	261,198,344
	HiTE	v3.2	31 MB	25:44:09	**12,813,196**
G. gallus	RepeatScout	v1.0.5	NA	NA	NA
	EDTA (sensitive)	v2.0.1	9.6 MB	25:27:10	4,692,748
	RepeatModeler2	v2.0.1	674 KB	16:47:46	17,221,388
	EarlGrey	v3.0	2.7 MB	66:00:32	62,572,832
	HiTE	v3.2	626 KB	**4:31:16**	**3,513,716**
T. guttata	RepeatScout	v1.0.5	NA	NA	NA
	EDTA (sensitive)	v2.0.1	13 MB	30:16:00	16,193,228
	RepeatModeler2	v2.0.1	772 KB	17:38:40	12,085,780
	EarlGrey	v3.0	1.5 MB	68:58:21	59,882,329
	HiTE	v3.2	639 KB	**3:45:14**	**2,640,940**
M. musculus	RepeatScout	v1.0.5	NA	NA	NA
	EDTA (sensitive)	v2.0.1	12 MB	49:22:36	36,687,352
	RepeatModeler2	v2.0.1	1.7 MB	16:22:25	31,525,268
	EarlGrey	v3.0	5.0 MB	70:27:40	64,618,280
	HiTE	v3.2	3.0 MB	**10:40:33**	**6,859,376**

Supplementary Table 14 Details of tools and parameters used. RS: RepeatScout; RM2: RepeatModeler2; CHE: CD-HIT-EST; LFP: LTR_FINDER_parallel; LHP: LTR_Harvest_parallel; TRF: Tandem Repeats Finder; RM: RepeatMasker; BM_EDTA: Benchmarking method of EDTA; BM_RM2: Benchmarking method of RepeatModeler2; BM_HiTE: Benchmarking method of HiTE.

Tools	Versions	Commands
RS	1.0.5	**(1)** build_lmer_table -sequence assembly -freq frequency<br>**(2)** RepeatScout -sequence assembly -freq frequency -output families
RM2	2.0.1	**(1)** BuildDatabase -name db_name assembly<br>**(2)** RepeatModeler -database db_name -pa threads -LTRStruct
EDTA	2.0.1	**(1)** perl EDTA.pl --genome assembly --threads threads --sensitive 1
HiTE	3.1.2	**(1)** python main.py --genome assembly --thread threads --outdir out_dir --chunk_size size --plant [1/0]
EarlGrey	3.0	**(1)** earlGrey -g assembly -s species_name -o out_dir -t threads
RMBlast	2.9.0	**(1)** makeblastdb -dbtype nucl -in sequences<br>**(2)** blastn -db subject_path -query query_path -outfmt 6 > output
CHE	4.8.1	**(1)** cd-hit-est -aS 0.95 -aL 0.95 -c 0.8 -G 0 -g 1 -A 80 -i input -o output -T 0 -M 0
TRF	4.0.9	**(1)** TRF_PATH input 2 7 7 80 10 50 500 -f -d -m
LFP	1.2	**(1)** perl LTR_FINDER_parallel -harvest_out -seq assembly -threads threads
LHP	1.1	**(1)** perl LTR_HARVEST_parallel -seq assembly -threads threads
LTR_retriever	2.9.0	**(1)** LTR_retriever -genome assembly -inharvest scn_file -noanno -threads threads -u miu
EAHelitron	1.54	**(1)** perl EAHelitron -o prefix -u 20000 -T "*ATC*" -r 3 input
itrsearch	TE Finder 2.31	**(1)** itrsearch -i 0.7 -l 7 input
BM_RM2	RM 4.1.1	**(1)** RepeatMasker -lib repbase_lib -nolow -pa threads test_lib<br>**(2)** sh get_family_summary_paper.sh lib.out
BM_EDTA	RM 4.1.1<br>EDTA 2.0.1	**(1)** RepeatMasker -e ncbi -pa threads -q -no_is -norna -nolow -div 40 -lib [repbase_lib/test_lib] -cutoff 225 assembly<br>**(2)** perl lib-test.pl -genome assembly -std repbase_lib.out -tst test_lib.out -cat total
BM_HiTE	HiTE 3.2	**(1)** cd HiTE && python module/lib_evaluation.py -g assembly --standard_lib repbase_lib --test_lib test_lib --work_dir out_dir --thread threads --coverage_threshold [0.8/0.95/0.99] --cat Total

Supplementary Notes

Supplementary Note 1 Comparative analysis of HiTE library with other common TE libraries

Method: To comprehensively evaluate the advantages and limitations of the HiTE library in comparison to other commonly used TE libraries, we have conducted a thorough comparative study in *A. thaliana*, employing four widely utilized TE libraries: Repbase, RepeatMasker, EDTA, and RepeatModeler2. The RepeatMasker library is extracted from the RepeatMasker database using the command "python famdb.py families --format fasta_name -ad --curated '*Arabidopsis thaliana*'". Both EDTA and RepeatModeler2 are run with default parameters.

Results: By applying CD-HIT-EST with parameters "-aS 0.8 -aL 0.8 -c 0.8 -G 0 -g 1 -A 80", we reveal a total of 152 unique TE families in HiTE, 350 TE families shared with other libraries, and 1376 TE families that were missing in HiTE. Excluding the EDTA library from the analysis, HiTE exhibits 234 unique TE families, 268 shared TE families, and 793 missing TE families. Moreover, when compared solely with the Repbase library, HiTE identifies 257 unique TE families, 245 shared TE families, and 568 missing TE families, consistent with the *Not_found* results of BM_RM2 (Supplementary Table 2).

Cases: To further illustrate our findings regarding the unique, missing, and shared TE families of HiTE, we provide specific examples:

(1) Unique TE families in HiTE: Most of the unique TE families identified in the HiTE library exhibit relatively low copy numbers. Through multiple sequence alignment, we observe clear terminal inverted repeats (TIRs) and target site duplications (TSDs) within these families (Supplementary Fig. 17a). The copies within the TE boundaries show high homology, while the sequences outside the boundaries appear random. This indicates the authenticity of these unique TE families as true transposons, albeit their low copy numbers hinder their detection.

We also identify a unique family that shares significant similarity with Repbase sequences. An example is shown in Supplementary Fig. 17b, where HiTE successfully identifies an 8,644-bp DNA/CMC-Enspm transposon (labeled as N_151) with only two full-length copies, while the remaining copies are truncated. In contrast, RepeatModeler2 and EDTA can only annotate fragmented sequences. Both RepeatMasker and Repbase annotate the transposon ATENSPM5 as two separate segments. However, upon performing BLASTN alignments between N_151 and ATENSPM5, the two sequences can be accurately matched in segments, indicating a high degree of similarity. The inability of the alignment tools to recognize them as the same family can be attributed to significant local variations between the two sequences. These variations result in the misalignment of certain regions, which prevents them from being assigned to the shared TE families.

(2) Missing TE families in HiTE: For TIR transposons, the families that HiTE lacked are due to the absence of two or more full-length copies in the genome. Supplementary Fig. 18a presents an example where HiTE has failed to capture a transposon, ATENSPM12, annotated by Repbase and RepeatMasker. Multiple sequence alignment reveals that this transposon possesses only a single full-length copy. Furthermore, RepeatModeler2 and EDTA erroneously annotate satellite repeats, while HiTE focuses exclusively on TE identification and does not annotate satellites.

In the case of Helitron transposons, the missing families in HiTE are attributed to the inability of HelitronScanner to identify candidate Helitron elements. As shown in Supplementary Fig. 18b, HiTE has failed to identify a structurally intact Helitron element with multiple copies. However, when this element is inputted into the false-positive filtering module of HiTE, the algorithm accurately determines the TE boundaries, confirming it as a genuine transposon. Further examination reveals that HelitronScanner has incorrectly

identified a longer sequence containing this transposon. Thus, the Helitron identification module of HiTE can be enhanced with improved accuracy of HelitronScanner. Additionally, RepeatModeler2 annotates multiple fragmented sequences, and the 5' end recognized by EDTA deviates from the actual boundaries.

(3) Shared TE families between HiTE and other libraries: Supplementary Fig. 19 shows the identification of shared family sequences between HiTE and Repbase. For shared TIR transposons, HiTE and Repbase recognize identical TE boundaries, while RepeatMasker, RepeatModeler2, and EDTA can only annotate fragmented sequences (Supplementary Fig. 19a). Regarding shared Helitron transposons, HiTE, Repbase, and RepeatMasker identify the same TE boundaries, while RepeatModeler2 can only annotate fragmented sequences. EDTA appears to annotate the correct boundaries as well. However, upon closer inspection, the annotation only pertained to a partial segment of the TE family it recognized (Supplementary Fig. 19b).

In conclusion, HiTE has the capability to identify novel TE families that are not included by the other four commonly used TE libraries. The authenticity of these families is validated through multiple sequence alignments using copies obtained from the genome. HiTE also exhibits fewer fragmented annotations compared to RepeatModeler2 and EDTA. However, HiTE may miss TE families lacking more than two full-length copies due to the challenge of reliably determining TE boundaries.

Supplementary Note 2 Comparing benchmarking methods for EDTA, RepeatModeler2, and HiTE

For convenience, we refer to the benchmarking methods of EDTA, RepeatModeler2, and HiTE as BM_EDTA, BM_RM2, and BM_HiTE, respectively. The BM_EDTA involves comparing the test annotation to existing curated genome annotations and calculating true positives (TP) and false negatives (FN). However, even when we fragment TE sequences within the test library, we still obtain consistent high performance with full-length TE libraries. This indicates that BM_EDTA cannot exclude the influence of fragmented sequences, potentially leading to misleadingly high sensitivity.

The BM_RM2 aligns the tested TE library with the gold standard library and categorizes them into four levels: "*Perfect*", "*Good*", "*Present*", and "*Not found*". "*Perfect*" families are those for which one sequence in the tested library matches with >95% sequence similarity and >95% length coverage to a family consensus in the gold standard library. "*Good*" families are those in which multiple overlapping sequences in the tested library match with >95% similarity and >95% coverage to the curated consensus. A family is considered "*Present*" if one or multiple library sequences align with >80% similarity and >80% coverage to the reference consensus sequence. Below these thresholds, a family is considered "*Not found*". Unlike BM_EDTA, BM_RM2 takes the integrity of the sequence into account. Full-length TE families typically receive a perfect level, while fragments may only get a good, present, or even not found level. However, BM_RM2 does not present the false positive rates in the tested TE library.

To better assess the quality of the test library, we have designed a new evaluation approach, referred to as BM_HiTE, as shown in Supplementary Fig. 1. An ideal TE library should exclusively consist of intact TE families, without any fragments. However, the genome contains many fragmented copies of full-length TEs, and their proportion even surpasses that of intact full-length TEs. The fragmented TE sequences in the test library can align to these fragmented copies of full-length TEs, causing misleadingly high sensitivity. Therefore, to accurately assess the quality of the test library, we retain only the intact full-length TE copies. However, even within the same TE family, TE instances can exhibit divergences, leading to fragmented alignments when using BLASTN for alignment. To retrieve full-length copies of TEs, we use our FMEA approach to concatenate BLASTN alignment results, setting the gap threshold for fragment concatenation to *TE_length* * *full_length_coverage*. Here, *TE_length* represents the length of the TE consensus sequence, and

full_length_coverage denotes the coverage threshold for full-length copies, such as 0.8.

The major difference between BM_HiTE and BM_EDTA lies in the criteria for calculating true positives. BM_EDTA considers any sequence that has any length of match with the standard library as a true positive. In BM_HiTE, a sequence is considered a true positive only when the length of the overlap between the test and Repbase TE sequence exceeds a threshold, such as 80% of their lengths respectively. As shown in Supplementary Fig. 1, all others are considered as false positives, including alignments with significant shifts, longer sequences containing the true TE, and fragmented TE sequences. BM_HiTE mitigates the influence of fragmented sequences on the results, making it more suitable for evaluating high-quality full-length TE libraries.

Supplementary Note 3 An illustration of how HiTE adjusts the boundaries for TIR elements

The non-homologous sequences should be present outside the boundaries of TEs. However, as shown in Supplementary Fig. 13, the coarse-grained boundaries of TEs detected by the HiTE-FMEA algorithm are not the true boundaries and contain many non-homologous bases. To address this issue, we extend both ends of the candidate TE copies and align them using multiple sequence alignment. Subsequently, a 10-bp sliding window is used to detect homology boundaries in the alignments.

For example, taking the starting boundary of 5' as a reference, the sliding window is moved to the right. We then calculate the homology ratio of each column in the sliding window and determine the average homology ratio of all columns in the sliding window. The sliding window with an average homology ratio above the threshold is identified as the homology window, and the first column of the window is considered the homology boundary. Moreover, to ensure that all the left sides of the homology boundary are non-homology columns, the sliding window is moved to the left from the homology boundary. The same approach is applied to determine the ending boundary of the 3' end. After identifying the homologous boundaries, we assess whether the candidate transposons possess specific transposon structures, such as TSD and terminal inverted repeat structures for TIR transposons.

Supplementary Note 4 Experimental result analysis of the filtering method

The genome contains many fragmented copies of full-length TEs, and their proportion even surpasses that of intact full-length TEs. The fragmented TE sequences in the test library can be aligned to these fragmented copies of full-length TEs, causing misleadingly high sensitivity. Therefore, when HiTE filters out numerous fragmented sequences, it exhibits lower sensitivity compared to HiTE-NoFiltering using BM_EDTA (Supplementary Table 6).

To address this issue, we have designed a new evaluation approach, referred to as BM_HiTE, as shown in Supplementary Fig. 1. We retain only the intact full-length TE copies. Only when the length of the overlap between the test and Repbase TE sequence exceeds a threshold, such as 80% of their lengths respectively, it is considered a true positive. All others are considered as false positives, including alignments with significant shifts, longer sequences containing the true TE, and fragmented TE sequences.

As shown in Supplementary Table 5, our filtering method results in a reduction in the number of perfect TE families identified by HiTE. This indicates that some genuine transposons are filtered out due to the inability to

locate their homologous boundaries using multiple sequence alignment. Most of these are low-copy-number transposons, requiring manual expert efforts to determine their authenticity. Supplementary Tables 7 and 8 demonstrate that our filtering method significantly improves precision while slightly sacrificing sensitivity, leading to a substantial reduction in false positives.

In summary, although the filtering method of HiTE filters out many false positive sequences, it may miss some genuine low-copy-number TEs. However, its feature of dynamically adjusting boundaries allows many TE instances to have precise boundaries. Compared to EDTA, the filtering method of HiTE shows higher performance.

Supplementary Note 5 Example description of Fault-tolerant Mapping Expansion Algorithm (FMEA)

Due to the existence of insertion, deletion, and multiple TE sequences, multiple subsequence alignments will be generated in the candidate repeat area, as shown in manuscript Fig. 3a: ***a, b, c, d, e, f***. The algorithm used for this process can be described as follows:

(1) The process begins by setting an extended threshold value, ***p***, and sorting the alignments by their starting and ending positions.

(2) For each alignment, we determine whether its adjacent alignment can expand the length of the sequence. For example, consider the alignment of subsequence ***a***, whose starting and ending positions are s_1 and e_1, and the starting and ending positions of subsequence ***b*** are s_2 and e_2. If $e_2 > e_1$ and $s_2 - e_1 < p$, it indicates that adding ***b*** can expand the length of the current subsequence. Thus, we connect the subsequences ***a*** and ***b***. Similarly, we connect subsequences ***c*** and ***d***. However, if $s_5 - e_4 > p$, it means that the subsequence ***e*** is too far from subsequence ***d*** to cross the gap in the middle, and they should belong to two different TE instances. In the example provided, the candidate TE instances are TE sequence 1 (starting s_1, terminating e_4, composed of subsequences ***a***, ***b***, ***c***, and ***d***) and TE sequence 2 (starting s_5, terminating e_6, corresponding to subsequences ***e*** and ***f***).

(3) Since the query sequence may be aligned to multiple different target sequences, a set with overlapping sequences will be obtained. If two sequences in the overlapped set have more than 95% overlap, they are considered copies of each other. By selecting the longest sequence as the representative sequence, we obtain a collection of non-overlapping repeats.

Supplementary Note 6 The main differences between HiTE and existing methods

The de novo method, signature-based method, and filtering method are three common approaches in the current TE identification/annotation pipeline, used by tools like HiTE, RepeatModeler2, EDTA, and EarlGrey. However, HiTE distinguishes itself from existing pipelines in the design and implementation of these three methods:

The de novo method: RepeatModeler2 employs RECON and RepeatScout for the identification of TE families. While EDTA primarily relies on the signature-based method for identifying LTR, TIR, and Helitron transposons, it employs the sequence repetition feature of RepeatModeler2 to detect non-LTR and unclassified transposons that may be missed by signature-based methods. EarlGrey employs a "BLAST, Extract, Extend (BEE)" strategy based on the results from RepeatModeler2, with the goal of generating maximum-length de novo TE consensus sequences. In contrast to the existing pipeline, HiTE develops a novel fault-tolerant mapping expansion algorithm, denoted as FMEA, to identify the full-length TEs.

As shown in Supplementary Fig. 2a, methods such as RECON have been used to cluster alignments,

potentially leading to the creation of fragmented TE families due to the excessive fragmentation in alignments. In contrast, our FMEA algorithm is not primarily concerned with identifying precise TE families. Instead, it is designed to connect and span insertions and gaps between alignment segments, aiming to preserve the integrity of TE instances as much as possible.

The FEMA algorithm is the de novo TE searching module of HiTE, which provides the input for the subsequent signature-based TE searching module. As shown in Supplementary Table 2, the FMEA algorithm outperforms other pipelines in identifying more full-length TE families.

The signature-based method: We elucidate the differences between HiTE and existing pipelines in the identification of four types of transposons: LTR, TIR, Helitron, and non-LTR. To our knowledge, neither RepeatModeler2 nor EarlGrey has designed signature-based modules specifically for TIR, Helitron, and non-LTR transposons.

(i) For the identification of LTR transposons, HiTE, EDTA, and RepeatModeler2 use the LTR_retriever, whereas EarlGrey relies on LTR_Finder and RepeatCrat[1]. EDTA retains the raw results from LTR_retriever. In contrast, both HiTE and RepeatModeler2 develop different methods to reduce redundancy, a necessity due to the frequent deletions within LTR transposons. RepeatModeler2 uses MAFFT and Ninja[2] to treat gaps as a single difference, then uses Refiner[3] to generate consensus sequences. On the other hand, HiTE begins with BLASTN for all-vs-all comparisons, applies our FMEA algorithm to bridge gaps, clusters sequences that bridge gaps, performs multiple sequence alignment using MAFFT, and ultimately leads to the generation of consensus sequences based on the majority rule.

(ii) For the identification of TIR and Helitron transposons, both HiTE and EDTA have developed separate modules to detect these two types of transposons. EDTA employs TIR-learner for identifying TIR transposons and utilizes HelitronScanner for identifying Helitron transposons. In contrast, HiTE has developed the HiTE-TIR and HiTE-Helitron modules tailored for these two types of transposons. Unlike EDTA, which uses the whole genome as input, HiTE performs identification on all potential TE candidate fragments generated by our FMEA algorithm. As shown in Supplementary Fig. 2b, given the misleading nature of TE structural signals, the signature-based methods using the whole genome as input often lead to more false positives.

As shown in Supplementary Table 5, under the same conditions without the use of filtering algorithms, the signature-based method of HiTE (HiTE-NoFiltering) can identify more perfect TE families compared to the signature-based method of EDTA (EDTA-NoFiltering) when using the benchmarking method of RepeatModeler2 (BM_RM2).

(iii) For the identification of non-LTR transposons, reliable de novo identification of non-LTR elements is difficult due to their inherently challenging recognition characteristics. EDTA does not incorporate a signature-based module for identifying non-LTR transposons. Instead, it uses the sequence repetition feature of RepeatModeler2 for non-LTR transposon identification. We have developed the HiTE-NonLTR module specifically designed for identifying non-LTR transposons, which searches for target site duplications (TSDs) and polyA tails for non-LTR transposons based on the outputs of our FMEA algorithm. Candidate non-LTR elements are then aligned to the genome to obtain their full-length copies, followed by multiple sequence alignment of all copies. Next, we have designed a dynamic boundary adjustment method to search for the homologous boundaries of the copies, determining the raw 5’ and 3’ ends. Each copy is then examined to identify the polyA tail near the raw 3’ end, establishing the true 3’ end of each copy. With the 3’ end established, all possible 3’ end TSDs are obtained, and corresponding 5’ end TSDs are searched near the raw 5’ end to determine the true 5’ end of each copy. To minimize false positives, we require that a genuine non-LTR element must have a copy count with TSDs greater than half of all copy counts, or exceed 5. While this stringent filtering method ensures reliability in identification, it may result in the loss of some intact LINE elements with low copy

numbers. Considering the conservative nature of domains across various TEs, we use a curated LINE domain library to retain candidate LINE elements harboring intact domains. The curated LINE domain library is derived from the RepeatPeps.lib within RepeatMasker, which is also used in the classification module of RepeatModeler2. HiTE-NonLTR exhibits excellent performance on species abundant in non-LTR elements, such as mouse and Drosophila. However, its performance still needs enhancement on species with limited non-LTR elements, such as Arabidopsis and rice (Supplementary Tables 9-11).

Due to the variability and lack of discernible structural signals of non-LTR elements, we have also developed a homology-based non-LTR searching module called HiTE-NonLTR-homology to achieve high-precision non-LTR annotation (Supplementary Tables 9-11).

The filtering method: RepeatModeler2 uses LTR_retriever to identify LTR transposons, which play a pivotal role as the primary reference during the integration of results from RECON and RepeatScout. Furthermore, RepeatModeler2 makes use of the parameter '*-LTRMaxSeqLen*' to set a limit on the maximum length of LTR internal sequences. Additionally, RepeatModeler2 employs TRF to mask tandem repeats. EarlGrey employs RepeatMasker with a conservative score threshold of 400 ('*-cutoff 400*') to eliminate weak matches that are less likely to represent genuine TE sequences. Additionally, any TE annotations shorter than 100 base pairs are discarded. As shown in Supplementary Figs. 3 and 4, both EDTA and HiTE use various filtering methods. Among these methods, a flanking sequence-based filtering method employed by EDTA, LTR_retriever, and HiTE is particularly crucial (Supplementary Fig. 5).

As shown in Supplementary Fig. 2c, the flanking sequence-based filtering method of HiTE differs from those of EDTA and LTR_retriever in that we have developed a novel homology-based filtering method based on multiple sequence alignment. This method not only filters out many false-positive sequences but also dynamically identifies genuine TE boundaries. As shown in Supplementary Tables 5-7, the filtering method of HiTE shows a substantial superiority over that of EDTA. The filtering method is based on the following concept: although it exhibits a bias, it is generally assumed that transposons are inserted randomly into the genome. Therefore, collecting all full-length copies of a transposon and subjecting them to a multiple sequence alignment reveals that outside the transposon boundaries, the alignment region displays randomness. In contrast, within the transposon boundaries, the alignment region exhibits a high degree of consistency since all are copies of the same transposon.

In summary, we modularized our method, where each module can be used independently as a specific TE identification tool.

Supplementary Note 7 Contribution of TEs to genome size

The amplification or contraction of TEs, affected by environmental stressors, is closely related to the genome size[4,5]. LTR retrotransposons, especially the Ty3-gypsy elements, which are the major component in most plants, play an important role in the genome size variation across the Oryza genus. By applying HiTE to several common rice subspecies, *Oryza sativa*, *Oryza rufipogon*, and *Oryza glaberrima*, we observed that there is significant genome size variation among these rice genus, and the main source of genome size difference is the Gypsy transposon (Supplementary Fig. 9), as previously documented[6]. The TE libraries of rice genomes are generated by HiTE using the default parameters, and RepeatMasker is then used to generate the length coverage based on these TE libraries.

Supplementary Note 8 Influence of parameters on HiTE

Most of the parameters in HiTE have been optimized and require no further adjustments. To assess the influence of parameter on HiTE, we select the two critical parameters for testing: *chunk_size*, and *flanking_len*. The *chunk_size* refers to cutting the genome into blocks of equal size, and the *flanking_len* is used to extend the candidate TEs to search for valid TSDs. Since these parameters do not affect the identification of LTR elements, which are detected by LTR_retriever, we use *C. briggsae* as the test species, as it contains few LTR elements.

The impact of *chunk_size* on performance fluctuates. Due to the diverse distribution of TEs across genomes of different species, setting *chunk_size* to an optimal value is challenging. However, slicing the genome may lead to the loss of some low-copy and scattered TEs, resulting in a decrease in perfect TE families (Supplementary Fig. 10a). Considering larger *chunk_size* requires increased computational resources, HiTE sets *chunk_size* to 400 MB by default.

Disabling the fine-grained boundary identification module by setting *flanking_len* to 0 results in the lowest performance (Supplementary Fig. 10b). This indicates that the majority of TEs identified in the FMEA algorithm possess coarse-grained boundaries, while setting *flanking_len* to 10 reveals the actual boundaries of most TEs. This suggests that the distance between the rough and real boundaries is not substantial. A larger *flanking_len* helps to detect more genuine TEs, albeit with increased computational costs. Therefore, HiTE sets *flanking_len* to 50 by default.

Supplementary Note 9 Running time of HiTE

We evaluated the running time and memory of HiTE based on *O. sativa*, *D. melanogaster*, *D. rerio*, *C. briggsae*, *Z. mays*, *A. thaliana, G. gallus, T. guttata, and M. musculus*. The evaluation was conducted on a server with 40 CPU cores (Intel(R) Xeon(R) CPU E5-2630 v4 @ 2.20GHz) and 256 GB RAM, and the results are presented in Supplementary Table 13.

Supplementary Note 10 The Impact of tandem repeats on HiTE

HiTE is currently unable to identify LTRs with large tandem repeats: Typically, tandem repeats are masked before conducting TE identification and annotation, which can eliminate false positives caused by tandem repeats and reduce runtime. However, the detection of LTR transposons in HiTE uses the genome without pre-masking tandem repeats, as shown in Fig. 1h of the main text. Our current LTR identification module is based on a pipeline composed of LTR_harvest, LTR_finder, and LTR_retriever, which has been applied to multiple tools, including EDTA and RepeatModeler2.

The Dasheng LTR in rice is a long non-autonomous transposon that does not encode proteins[7]. Our analysis reveals that while this transposon is identified by LTR_finder, it is filtered out by LTR_retriever, which currently filters out LTR elements containing large tandem repeats. Therefore, this constraint will be introduced by any TE identification tool using LTR_retriever.

HiTE can identify the TE families within the centromeric/telomeric regions: Naish et al. generated a T2T *Arabidopsis thaliana* genome and observed multiple insertions of the ATHILA family within the centromeric tandem repeat region CEN180[8]. We pinpointed the ATHILA5 transposon positioned at Chr5: 12739578-12750538, and HiTE successfully identifies this transposon, as CEN180 is only present in a small portion of the 5' LTR. Similarly, we examined CRM in maizes[9], which itself does not harbor large tandem repeats. Consequently, HiTE can also detect CRM transposons.

Therefore, although LTR_retriever filters out LTRs containing internal large tandem repeats, it can still identify elements like ATHILA and CRM. These elements, though inserted into the telomeres or centromeres,

do not possess large tandem repeats themselves.

HiTE can identify other types of TEs containing large tandem repeats: In addition to LTRs, we also examined other TE identification modules within HiTE and found that HiTE can identify transposons containing large tandem repeats. As shown in Fig. 1d of the manuscript, following pre-masking of tandem repeats, we have developed a de novo TE searching method named HiTE-FMEA, which enables the bridging of large gaps in alignments. Since our de novo identification of TIR and Helitron transposons uses the coarse-grained TE results identified by HiTE-FMEA, HiTE can identify TIR and Helitron transposons containing internal tandem repeats. For example, there exists an 11 kb TIR element in rice, located at chr1:3748476-3759610, harboring large tandem repeats internally. Through the implementation of the HiTE-FMEA algorithm, HiTE demonstrates the capability to detect the full-length transposon.

Assessing the Impact of T2T Genome on HiTE in comparison to genomes with gaps: Compared to existing genomes, T2T genomes typically address the assembly of complex regions such as telomeres and centromeres. We used T2T assemblies of *Arabidopsis thaliana*[8] and *rice*[10] to evaluate the influence of employing T2T assemblies on HiTE performance. As shown in Supplementary Table 12, although T2T assemblies do not exhibit a significant increase in the number of perfect families identified in HiTE compared to assemblies with gaps, they do show notable performance improvements in BM_HiTE across different threshold evaluations, particularly with stricter thresholds like 95% and 99%. This indicates that the use of T2T assemblies can yield favorable TE annotation results.

Supplementary References

1 Wong, W. Y. & Simakov, O. RepeatCraft: a meta-pipeline for repetitive element de-fragmentation and annotation. *Bioinformatics* **35**, 1051-1052 (2019).

2 Wheeler, T. J. in *Algorithms in Bioinformatics: 9th International Workshop, WABI 2009, Philadelphia, PA, USA, September 12-13, 2009. Proceedings 9.* 375-389 (Springer).

3 Hubley, R., Wheeler, T. J. & Smit, A. F. Accuracy of multiple sequence alignment methods in the reconstruction of transposable element families. *NAR genomics and bioinformatics* **4**, lqac040 (2022).

4 Canapa, A., Barucca, M., Biscotti, M. A., Forconi, M. & Olmo, E. Transposons, genome size, and evolutionary insights in animals. *Cytogenetic and genome research* **147**, 217-239 (2015).

5 Zhang, X. & Qi, Y. The landscape of Copia and Gypsy retrotransposon during maize domestication and improvement. *Frontiers in Plant Science* **10**, 1533 (2019).

6 Dai, S.-f. *et al.* Genome size variation and evolution driven by transposable elements in the genus oryza. *Frontiers in Plant Science* **13** (2022).

7 Jiang, N. *et al.* Dasheng: a recently amplified nonautonomous long terminal repeat element that is a major component of pericentromeric regions in rice. *Genetics* **161**, 1293-1305 (2002).

8 Naish, M. *et al.* The genetic and epigenetic landscape of the Arabidopsis centromeres. *Science* **374**, eabi7489 (2021).

9 Chen, J. *et al.* A complete telomere-to-telomere assembly of the maize genome. *Nature genetics* **55**, 1221-1231 (2023).

10 Shang, L. *et al.* A complete assembly of the rice Nipponbare reference genome. *Molecular Plant* **16**, 1232-1236 (2023).
