# Supplementary material for: HiTE: a fast and accurate dynamic boundary adjustment approach for full-length transposable element detection and annotation
Source: Nat Commun. 2024 Jul 2;15:5573. doi: 10.1038/s41467-024-49912-8 (PMC11219922; doi:10.1038/s41467-024-49912-8)
Supplement: Supplementary file 3 — Description of Additional Supplementary Files [file 41467_2024_49912_MOESM3_ESM.pdf]

Description of Additional Supplementary Files

File Name: Supplementary Data 1
Description: The information of novel TIR transposons identified by HiTE in rice.

File Name: Supplementary Data 2
Description: The information of eight well-known TIR transposons and their annotation status in five different TE libraries. "NA" represents fragmented annotation and therefore its sequence length is not included in the statistics. All TE libraries can be downloaded from https://github.com/CSU-KangHu/TE_annotation.
