# Supplementary material for: HiTE: a fast and accurate dynamic boundary adjustment approach for full-length transposable element detection and annotation
Source: Nat Commun. 2024 Jul 2;15:5573. doi: 10.1038/s41467-024-49912-8 (PMC11219922; doi:10.1038/s41467-024-49912-8)
Supplement: Supplementary file 6 — Reporting Summary [file 41467_2024_49912_MOESM6_ESM.pdf]

nature portfolio

Corresponding author(s): Bin Hu, Jianxin Wang

Last updated by author(s): Jun 22, 2024

Reporting Summary

Nature Portfolio wishes to improve the reproducibility of the work that we publish. This form provides structure for consistency and transparency in reporting. For further information on Nature Portfolio policies, see our Editorial Policies and the Editorial Policy Checklist.

Statistics

For all statistical analyses, confirm that the following items are present in the figure legend, table legend, main text, or Methods section.

n/a	Confirmed	
☐	☒	The exact sample size (n) for each experimental group/condition, given as a discrete number and unit of measurement
☒	☐	A statement on whether measurements were taken from distinct samples or whether the same sample was measured repeatedly
☒	☐	The statistical test(s) used AND whether they are one- or two-sided<br>*Only common tests should be described solely by name; describe more complex techniques in the Methods section.*
☒	☐	A description of all covariates tested
☒	☐	A description of any assumptions or corrections, such as tests of normality and adjustment for multiple comparisons
☐	☒	A full description of the statistical parameters including central tendency (e.g. means) or other basic estimates (e.g. regression coefficient) AND variation (e.g. standard deviation) or associated estimates of uncertainty (e.g. confidence intervals)
☒	☐	For null hypothesis testing, the test statistic (e.g. F, t, r) with confidence intervals, effect sizes, degrees of freedom and P value noted<br>*Give P values as exact values whenever suitable.*
☒	☐	For Bayesian analysis, information on the choice of priors and Markov chain Monte Carlo settings
☒	☐	For hierarchical and complex designs, identification of the appropriate level for tests and full reporting of outcomes
☒	☐	Estimates of effect sizes (e.g. Cohen's d, Pearson's r), indicating how they were calculated

Our web collection on statistics for biologists contains articles on many of the points above.

Software and code

Policy information about availability of computer code

Data collection	The RepeatMasker library was extracted from the RepeatMasker (version 4.1.1, http://www.repeatmasker.org) database using its "famdb.py" script.
Data analysis	This manuscript utilized open software and scripts described in the Methods section and Supplementary Table 14. We used RepeatScout (version 1.0.5), RepeatModeler (version 2.0.1), EDTA (version 2.0.1), EarlGrey (version 3.0), RMBlast (version 2.9.0), cd-hit-est (version 4.8.1), TRF (version 4.0.9), LTR_FINDER_parallel (version 1.2), LTR_HARVEST_parallel (version 1.1), LTR_retriever (version 2.9.0), EAHelitron (version 1.54), TE Finder (version 2.31), RepeatMasker (version 4.1.1), Python3, Mafft (version 7.520), Nextflow (version 22.10.6), HelitronScanner (version 1.1), and HiTE (version 3.1.2). HiTE is publicly available at GitHub [https://github.com/CSU-KangHu/HiTE].

For manuscripts utilizing custom algorithms or software that are central to the research but not yet described in published literature, software must be made available to editors and reviewers. We strongly encourage code deposition in a community repository (e.g. GitHub). See the Nature Portfolio guidelines for submitting code & software for further information.

Data

Policy information about availability of data

All manuscripts must include a data availability statement. This statement should provide the following information, where applicable:

- Accession codes, unique identifiers, or web links for publicly available datasets
- A description of any restrictions on data availability
- For clinical datasets or third party data, please ensure that the statement adheres to our policy

The reference genomes for nine species, including Oryza sativa (assembly IRGSP-1.0), Caenorhabditis briggsae (assembly CB4), Drosophila melanogaster (assembly Release 6 plus ISO1 MT), Danio rerio (assembly GRCz11), Zea mays (assembly Zm-B73-REFERENCE-NAM-5.0), Arabidopsis thaliana (assembly TAIR10.1), Gallus gallus (assembly GCF_000002315.5), Taeniopygia guttata (assembly GCF_000151805.1), and Mus musculus (assembly GCA_000001635.2), can be accessed through NCBI GenBank [https://www.ncbi.nlm.nih.gov/genome/]. The other rice genome (Oryza sativa L. ssp. japonica cv. "Nipponbare" v. MSU7) used in the Ghd2 gene experiment of this study, as well as its annotation with respect to both genes and repeats, can be accessed through the Rice Genome Annotation Project [http://rice.uga.edu/]. The telomere-to-telomere assembly of the maize, rice, and Arabidopsis genomes used in this study can be found in CyVerse [https://data.cyverse.org/dav-anon/iplant/home/laijs/Zm-Mo17-REFERENCE-CAU-2.0/], RiceSuperPIRdb [http://www.ricesuperpir.com/web/download], and GitHub [https://github.com/schatzlab/Col-CEN/tree/main/v1.2]. The curated TE libraries used in this study can be accessed through a paid subscription to Repbase [https://www.girinst.org/repbase/]. Additionally, the TE libraries and novel transposons generated in this study are publicly available in the GitHub repository CSU-KangHu/TE_annotation [https://github.com/CSU-KangHu/TE_annotation] and Zenodo.

Research involving human participants, their data, or biological material

Policy information about studies with human participants or human data. See also policy information about sex, gender (identity/presentation), and sexual orientation and race, ethnicity and racism.

Reporting on sex and gender	N/A
Reporting on race, ethnicity, or other socially relevant groupings	N/A
Population characteristics	N/A
Recruitment	N/A
Ethics oversight	N/A

Note that full information on the approval of the study protocol must also be provided in the manuscript.

Field-specific reporting

Please select the one below that is the best fit for your research. If you are not sure, read the appropriate sections before making your selection.

☒ Life sciences ☐ Behavioural & social sciences ☐ Ecological, evolutionary & environmental sciences

For a reference copy of the document with all sections, see nature.com/documents/nr-reporting-summary-flat.pdf

Life sciences study design

All studies must disclose on these points even when the disclosure is negative.

Sample size	All tests used genome assemblies from nine diverse species, including both model and non-model organisms, encompassing plants and animals. These species exhibit significant variations in genome size and TE abundance. As shown in Supplementary Table 1, these nine species span nearly all ranges of TE abundance in genomes. Additionally, these species cover a range of genome sizes from 104 MB to 2.6 GB.
Data exclusions	No data was excluded.
Replication	All experimental findings can be replicated following the instructions provided in the documentation: https://github.com/CSU-KangHu/HiTE/wiki/Experiment-reproduction.
Randomization	We randomly selected nine species with varying genome sizes and TE abundances to ensure HiTE exhibits robust generalization capabilities.
Blinding	We selected various species for experimental testing, including well-studied species such as rice and Drosophila, as well as less-studied species such as Gallus gallus and Taeniopygia guttata.

Reporting for specific materials, systems and methods

We require information from authors about some types of materials, experimental systems and methods used in many studies. Here, indicate whether each material, system or method listed is relevant to your study. If you are not sure if a list item applies to your research, read the appropriate section before selecting a response.

Materials & experimental systems

n/a	Involved in the study
☒	☐ Antibodies
☒	☐ Eukaryotic cell lines
☒	☐ Palaeontology and archaeology
☐	☒ Animals and other organisms
☒	☐ Clinical data
☒	☐ Dual use research of concern
☐	☒ Plants

Methods

n/a	Involved in the study
☒	☐ ChIP-seq
☒	☐ Flow cytometry
☒	☐ MRI-based neuroimaging

Animals and other research organisms

Policy information about studies involving animals; ARRIVE guidelines recommended for reporting animal research, and Sex and Gender in Research

Laboratory animals	The tests in this manuscript were conducted using publicly available assemblies of the animals Caenorhabditis briggsae, Drosophila melanogaster, Danio rerio, Gallus gallus, Taeniopygia guttata, and Mus musculus.
Wild animals	N/A
Reporting on sex	N/A
Field-collected samples	N/A
Ethics oversight	N/A

Note that full information on the approval of the study protocol must also be provided in the manuscript.

Dual use research of concern

Policy information about dual use research of concern

Hazards

Could the accidental, deliberate or reckless misuse of agents or technologies generated in the work, or the application of information presented in the manuscript, pose a threat to:

No	Yes
☒	☐ Public health
☒	☐ National security
☒	☐ Crops and/or livestock
☒	☐ Ecosystems
☒	☐ Any other significant area

Experiments of concern

Does the work involve any of these experiments of concern:

No	Yes
☒	☐ Demonstrate how to render a vaccine ineffective
☒	☐ Confer resistance to therapeutically useful antibiotics or antiviral agents
☒	☐ Enhance the virulence of a pathogen or render a nonpathogen virulent
☒	☐ Increase transmissibility of a pathogen
☒	☐ Alter the host range of a pathogen
☒	☐ Enable evasion of diagnostic/detection modalities
☒	☐ Enable the weaponization of a biological agent or toxin
☒	☐ Any other potentially harmful combination of experiments and agents
